# The Novel Secreted *Meloidogyne incognita* Effector MiISE6 Targets the Host Nucleus and Facilitates Parasitism in *Arabidopsis*

**DOI:** 10.3389/fpls.2018.00252

**Published:** 2018-03-23

**Authors:** Qianqian Shi, Zhenchuan Mao, Xiaoping Zhang, Jian Ling, Runmao Lin, Xi Zhang, Rui Liu, Yunsheng Wang, Yuhong Yang, Xinyue Cheng, Bingyan Xie

**Affiliations:** ^1^Institute of Vegetables and Flowers, Chinese Academy of Agricultural Sciences, Beijing, China; ^2^Department of Plant Pathology and Ministry of Agriculture Key Laboratory for Plant Pathology, China Agricultural University, Beijing, China; ^3^School of Medical Science, Chifeng University, Chifeng, China; ^4^College of Life Sciences, Beijing Normal University, Beijing, China

**Keywords:** *Meloidogyne incognita*, effector, nuclear localization signal (NLS), hypersensitive response (HR), plant–nematode interaction, comparative transcriptome analysis

## Abstract

*Meloidogyne incognita* is highly specialized parasite that interacts with host plants using a range of strategies. The effectors are synthesized in the esophageal glands and secreted into plant cells through a needle-like stylet during parasitism. In this study, based on RNA-seq and bioinformatics analysis, we predicted 110 putative *Meloidogyne incognita* effectors that contain nuclear localization signals (NLSs). Combining the *Burkholderia glumae*–pEDV based screening system with subcellular localization, from 20 randomly selected NLS effector candidates, we identified an effector MiISE6 that can effectively suppress *B. glumae*-induced cell death in *Nicotiana benthamiana*, targets to the nuclei of plant cells, and is highly expressed in early parasitic J2 stage. Sequence analysis showed that MiISE6 is a 157-amino acid peptide, with an OGFr_N domain and two NLS motifs. Hybridization *in situ* verified that *MiISE6* is expressed in the subventral esophageal glands. Yeast invertase secretion assay validated the function of the signal peptide harbored in MiISE6. Transgenic *Arabidopsis thaliana* plants expressing *MiISE6* become more susceptible to *M. incognita*. Inversely, the host-derived RNAi of *MiISE6* of the nematode can decrease its parasitism on host. Based on transcriptome analysis of the *MiISE6* transgenic *Arabidopsis* samples and the wild-type samples, we obtained 852 differentially expressed genes (DEGs). Integrating Gene Ontology (GO) and Kyoto Encyclopedia of Genes and Genomes (KEGG) enrichment analyses, we found that expression of *MiISE6* in *Arabidopsis* can suppress jasmonate signaling pathway. In addition, the expression of genes related to cell wall modification and the ubiquitination proteasome pathway also have detectable changes in the transgenic plants. Results from the present study suggest that *MiISE6* is involved in interaction between nematode-plant, and plays an important role during the early stages of parasitism by interfering multiple signaling pathways of plant. Moreover, we found homologs of MiISE6 in other sedentary nematodes, *Meloidogyne hapla* and *Globodera pallida*. Our experimental results provide evidence to decipher the molecular mechanisms underlying the manipulation of host immune defense responses by plant parasitic nematodes, and transcriptome data also provide useful information for further study nematode–plant interactions.

## Introduction

*Meloidogyne incognita*, the southern root knot nematode (RKN), is an economically important, sedentary nematode that infests roots of a wide range of crop plants (Alioto et al., 2015). During parasitism, RKNs establish a close relationship with their hosts and induce the formation of giant cells (GCs) that provide essential nutrition for nematode parasitic life stages. The formation of GCs are mediated through secretions (referred to as effectors) from the esophageal gland (dorsal and subventral) that are injected into root cells via the stylet, resulting in physiological and morphological changes in root cells (Abad et al., 2003). The esophageal glands of *M. incognita* are composed of two subventral gland (SvG) cells and one dorsal gland cell. The SvG cells are active in the pre-parasitic and parasitic stages, producing effectors required for root invasion. The dorsal gland cell is active during the sedentary stages, synthesizing proteins involved in feeding site development and maintenance (Davis et al., 2000). Once secreted from esophageal glands into root cells, effectors may be localized to different cellular compartments where they may assume diverse cellular functions to increase parasitism. The manipulation of host cellular functions, such as host transcription, chromatin remodeling, and immune responses, involves targeting of the host nucleus by secreted effectors (Quentin et al., 2013).

It is hypothesized that effector proteins participate in a diverse range of host processes during nematode parasitism. Nematode effectors work by interacting with host proteins or mimicking host proteins, and are capable of degrading and modifying plant cell walls (Smant et al., 1998); suppressing host defense responses; and targeting plant signaling pathways, such as those involved in the shikimate pathway and the ubiquitin-proteasome pathway (Chronis et al., 2013; Chen et al., 2015; Niu et al., 2016). In addition to triggering the development of GCs, effectors play a central role in suppressing host defense responses to facilitate infection (Haegeman et al., 2012).

A growing number of studies have revealed that some secreted effectors can target the host cell nucleus and manipulate essential host cellular processes such as transcription, chromatin remodeling, and histone modification (Kay et al., 2007; Clapier and Cairns, 2009). Many effectors contain nuclear localization signals (NLSs). The functional characterization of nuclear-targeted effectors from bacteria is well documented. For instance, the VirE2 of *Agrobacterium*, which contains a NLS, was involved in the transfer of T-DNA to the plant cell nucleus to modulate histone gene expression and exert infection (Tzfira et al., 2001; Citovsky et al., 2004). Another TAL effector AvrXa7 of *Xoo* can activate the accumulation of OsSWEET14, which can induce a sugar efflux to feed bacteria in the xylem and apoplasm (Chen et al., 2010). In *Meloidogyne* nematode, previous studies have identified one nuclear located effector, 7H08, through subcellular localization verification among 13 candidate NLS effectors that are expressed in the esophageal gland (Zhang et al., 2015). The *M. javanica* effector MJ-NULG1a was found to contain two NLSs, and immunolocalization analysis showed that MJ-NULG1a was localized in the nuclei of giant cells during nematode parasitism, and that it played a central role in *M. javanica* parasitism (Lin et al., 2013). In *Heterodera schachtii*, the NLS-containing effector 10A07 can localize to the nucleus and then specifically interact with the nuclear-localized transcription factor IAA16, leading to interference of auxin signaling. Overexpression of *H. schachtii* 10A07 in *Arabidopsis thaliana* increased sensitivity to *H. schachtii* infection (Hewezi et al., 2015). Mounting evidence suggests that nucleus-targeted effectors play important roles in weakening host resistance or enhancing pathogen virulence during plant–pathogen interactions. However, further studies on additional related effectors are required to elucidate the molecular mechanisms behind this process.

Benefits from advances in genomic, transcriptomic, and bioinformatic approaches, have led to progress in identification and functional characterization of some nematode effectors, but most effectors are still unidentified. In this study, by transcriptome analysis, we predict 110 candidate NLS effectors from *M. incognita*. Of 20 randomly selected effector candidates, three can strongly suppress *B. glumae*-caused fast, localized HR responses in non-host *N. benthamiana*. Two of these effectors are verified as localizing to the nucleus. Of which, *MiISE6* is highly expressed in the early parasitism stages, and has a functional signal peptide. Utilizing transgenic technology and host-derived RNAi approaches, in addition to comparative transcriptome analysis, we try to determine the roles of *MiISE6* in *M. incognita* parasitism.

## Materials and Methods

### Nematodes, Plants and Growth Conditions

*Meloidogyne incognita* nematodes were propagated on 3-week-old pepper (*Capsicum annuum*, Qiemen) plants in an artificial environment from a single female egg mass after isolated from the Sijiqing farm (Beijing, China). Three lines (Avir-1, Avir-2, and Avir-3) of the nematodes that each from a female egg mass were continually reproduced for 30 generations to form stable populations and then used for experiments. Infective, second stage juveniles were collected by hatching the handpicked egg masses at 26°C.

*Nicotiana benthamiana* was used for plant hypersensitive response (HR) assay and subcellular localization observation. *Arabidopsis thaliana* (Col-0 ecotype) was used for transgene, plant-nematode interaction and parasitism analyses. Both plants were cultivated in a growth chamber at 23°C.

### Secreted NLSs Effector Prediction

Nematode genomes and transcripts used to predict effector proteins containing nuclear localization signals (NLSs) and for sequence homologue analysis in were downloaded from the National Center for Biotechnology Information (NCBI) (Supplementary Table S1). Signal peptide predictions were performed using SignalP 4.1^1^, predisi^2^, and phobius^3^. Subcellular location of proteins was predicted by TargetP^4^, and the proteins located in mitochondria were removed. The prediction of transmembrane domain and NLS were performed using TMHMM^5^ and NucPred (Brameier et al., 2007) based on a sequence score ≥ 0.5. Domains were predicted in local PuTTY based on HMMER 3.1b1 (Wong et al., 2015) and the active conserved domains were blasted to the Pfam database (Finn et al., 2014). A phylogenetic analysis of homologues was carried out using the maximum likelihood method with MEGA 5 (Tamura et al., 2011).

### Cell Death Suppression

The suppression of cell death elicited by *B. glumae* (from a gift of Dr. Sun, Chinese Agricultural University) on *N. benthamiana* leaves was performed as described in previous study (Sharma et al., 2013). Candidate effectors and *GFP* coding sequences were cloned into the pEDV vector to generate pEDV::effector and pEDV::GFP constructs, respectively. All constructs were introduced into *B. glumae* by electroporation, and the transformed bacteria were then suspended in 0.9% NaCl at 600 nm (OD_600_ = 0.4). *Burkholderia glumae* cells carrying pEDV::effector were infiltrated into the right side of *N. benthamiana* leaves using a 1 ml syringe. As controls, *B. glumae* cells carrying the pEDV::GFP were infiltrated into the left side of *N. benthamiana* leaves. Three leaves on 5 plants were used for each biological replicate, and at least three independent experiments were performed. Plants were kept at 25°C. Cell death was observed at 4 days post-inoculation. All primers were synthesized by Sangon and were listed in Supplementary Table S2.

### Subcellular Localization

For subcellular localization assays, MiISE2^Δsp^, MiISE10^Δsp^, MiISE6^Δsp^, MiISE6^Δsp_Δ109_157^, MiISE6^Δsp_Δ33_108^ and MiISE6^Δsp_MΔ109_118^ were amplified by PCR from the *M. incognita* cDNA, using I-5^TM^ 2 × High-Fidelity Master Mix (MCLAB, United States). PCR products were digested with *Nco* I and *Spe* I and inserted into pCAMBIA1302 digested with the same enzymes. The resulting plasmids harbored the effector gene fused with the GFP gene. The constructs were transformed into *Agrobacterium tumefaciens* GV3101. After inoculation in LB followed by horizontal shaking for approximately 30 h, *A. tumefaciens* cultures were diluted to an OD_600_ of 0.8 using infiltration medium (10 mM MES, 10 mM MgCl_2_ and 0.1 mM AS). Leaves at the four-leaf stage of *N. benthamiana* were infiltrated with bacterial inocula using a 1 ml syringe with no needle. After 24–48 h, the infiltrated zones of the leaf were observed under a confocal microscope (Zeiss LSM700, Germany).

### Quantitative RT-PCR (q-PCR) Assays

RNA samples were extracted from 300 *M. incognita* at different life stages (pre-J2, parasitic (par)-J2, par-J3, par-J4 and female) for developmental expression analysis using RNAprep pure Tissue Kit (Tiangen, China). The 18S gene (Genbank accession no. U81578) was used as an internal control.

In order to confirm the results of RNA-seq analysis, *Arabidopsis* plants grow under same condition as those used for the RNA-seq analysis were collected for q-PCR assays. RNA samples were extracted from 100 mg frozen plant tissue using RNAprep pure Plant Kit (Tiangen, China). The Actin2 gene (AT3G18780) was used as an internal control.

Then, 1 μg RNA was used for cDNA synthesis using PrimeScript^TM^ RT reagent Kit with gDNA Eraser (Perfect Real Time) (Takara, Japan) following the manufacturer’s protocol. Each 25 μl reaction mixture was prepared using SYBR Premix Ex Taq^TM^||(TaKaRa, Japan) on a BIO-RAD CFX96 (BIO-RAD, United States) following the manufacturer’s protocol. Cycling conditions for quantitative PCR were: 95°C for 5 min and 40 cycles of 95°C for 30 s and 60°C for 30 s. Gene expression changes were calculated using the 2^-ΔΔCT^ method. At least three independent experiments were performed, with four technical replicates for each reaction.

### *In Situ* Hybridization

Parasitic stage J2s of *M. incognita* were processed for *in situ* hybridization to confirm the expression site of *MiISE6* as previously described (de Boer et al., 1998). Digoxigenin-labeled sense and antisense cDNA probes were synthesized using the primers DIG-MiISE6-F and DIG-MiISE6-R. Hybridization signals within the nematode were detected with alkaline phosphatase-conjugated anti-digoxigenin antibody and substrate, and observed with an Olympus IX53 microscope.

Fluorescence *in situ* hybridization was performed using a 28 bp specific cDNA probe (CTTTCTTCAATTCCTT TCATCAACATCC) whose 5′ end was labeled with fluorescein isothiocyanate (FITC, Sangon Biotech) as previously described with some modification (Moter and Gobel, 2000), and the hybridization signals were observed under a confocal microscope (Zeiss LSM700, Germany).

### Validation of the Functionality of Signal Peptide

We used the yeast secretion system based on the yeast signal sequence trap vector pSUC2T7M13ORI (Jacobs et al., 1997) to validate the function of MiISE6 signal peptide. PCR products were digested with *Eco*RI and *Xho*I and inserted into pSUC2T7M13ORI to generate pSUC2::MiISE6. The primer pairs used for PCR amplification were pSUC2_MiISE6_F and pSUC2_ MiISE6_R. The constructs were transformed into the invertase negative yeast strain YTK12 using the Frozen-EZ Yeast Transformation II kit (Zymo Research, United States). After transformation, yeast was plated on CMD-W (minus Trp) plates for 3 days. Single colonies of YTK12 were replicated onto raffinose-containing YPRAA plates (with raffinose instead of sucrose). Invertase enzymatic activity was detected with TTC as described by Oh et al. (2009).

### Generation of Transgenic *Arabidopsis* Plants

For *MiISE6* overexpression in *Arabidopsis*, the coding sequence of the *MiISE6* gene without signal peptide was cloned into the vector pEGAD, and for host-derived gene silencing, the coding sequence of MiISE6 was inserted into the pFGC5941 vector in both sense and antisense orientations. There was an CHSA intron between sense and antisense fragments. Both pEGAD vector and pFGC5941 vector habor the BASTA selectable marker gene. The transformation of *Arabidopsis* was performed as previously described (Martínez-Zapater and Salinas, 1998). Transformed T1 seeds were sown on soil and were screened by spraying the herbicide BASTA 2 weeks later to select for transgenic plants. The spraying was repeated three times. Homozygous T3 seeds were used in this research. The expression level of *MiISE6* in different transgenic line were confirmed by RT-PCR, RNAi transgenic lines was confirmed by checking the CHSA intron fragment using primer CHSA-F/CHSA-R.

### Nematode Infection Assays

Four-week-old *Arabidopsis* were inoculated using 400 J2 of *M. incognita* per plant. 42 days after inoculation, the number of females was counted.

At least 15 plants were used for each treatment, and three independent experiments were performed. Statistically significant differences between treatments were determined by independent samples *t*-test (*P* < 0.05) using SPSS.

### RNA Sequencing and Transcriptome Analysis

For *M. incognita* transcriptome: 50 mg of freshly collected nematodes were used for RNA extraction using TRIzol reagent (Invitrogen) following the manufacturer’s instructions. At least 20 mg of total RNA was sent to the Shenzhen Genomics Institute for Solexa sequencing (commercial service, Shenzhen China), and three independent replicates (Avir-1, Avir-2, and Avir-3) were sequenced. The full genome sequences and gene annotation of *M. incognita* were downloaded from Wormbase as a reference genome (Abad et al., 2008). The RNA-seq reads were mapped to the reference genome using Tophat and subsequently assembled using cufflinks (Trapnell et al., 2010). The expression abundance of each gene was estimated with fragments per kilobase of transcript per million fragments mapped (FPKM) (Anders et al., 2015).

For *A. thaliana* transcriptome: The 14-day transgenic *Arabidopsis* and WT *Arabidopsis* grown on Murashige and Skoog (MS) medium were used for RNA extraction. RNA samples were extracted from 100 mg frozen plant tissue using RNAprep pure Plant Kit (Tiangen, China). Library construction and RNA-Seq were performed at BerryGenomics (Beijing, China), and four independent replicates were sequenced. The cDNA libraries with average insert sizes of 280 bp were sequenced by Illumina technology. The adaptor sequences and low-quality reads were filtered from the raw data. Tophat2 tools were used to map mRNA reads to the *Arabidopsis* genome (Langdon, 2015), and htseq-count was then used to calculate the expected FPKM mapped (Anders et al., 2015), and the edgeR package in R was applied to identify DEGs of 2 groups of samples with a threshold criterion of (FDR < 0.01) and |log_2_FC|≥ 1. We used AgriGO^6^ and KOBAS (Xie et al., 2011) to perform GO functional enrichment analysis and KEGG enrichment analysis with default parameters.

## Results

### Transcriptome Sequence Assembly and Prediction of Effectors Containing Secreted Nuclear Localization Signals (NLSs)

In total, 8.22 billion clean base pairs were generated from three independent replicates of *M. incognita* wild type population, with Illumina/Solexa sequencing reads of ∼70x of *M. incognita* genome. Of these, 13398 genes (95.90% of total) were shared by the three replicates (Supplementary Figure S1); detailed information is shown in Supplementary Datasheet S1. By a series bioinformatic analysis, such as searching out the sequences with signal peptides and discarding the sequences with putative transmembrane-spanning regions, analyzing subcellular location and removing proteins located in mitochondria, NLS motif predicting, totally 110 potential NLS effectors are predicted from *M. incognita*, and they are taken as candidate effectors with putative immune function for further analysis. Detailed information is shown in Supplementary Datasheets S1, S2.

### Immune Suppression Studies on Putative Effectors

**FIGURE 1:**
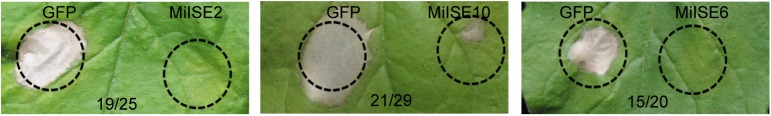
Putative *M. incognita* effectors suppress *B. glumae*-induced HR in *N. benthamiana*. The *N. benthamiana* leaves were photographed within 4 days after *B. glumae* inoculation to observe the cell death symptoms. The left half leaf sections were injected with *B. glumae* carrying pEDV::GFP and the right half sections were injected with *B. glumae* carrying pEDV::effector. The volume of bacteria solution injected in both side were equivalent. Numbers on the pictures, for example 19/25, indicate that 19 right leaf sections out of 25 inoculated leaves showed no or significantly less severe symptoms as compared with left sections.

Previous studies have shown that PPN effectors can suppress the plant hypersensitive response (HR) to facilitate parasitism (Niu et al., 2016; Chen et al., 2017). Here we used a pEDV-based type III secretion system (T3SS) in which *B. glumae* is used to deliver effectors to plant cells to verify the function of 20 randomly selected novel effector candidates in plant immune suppression. *Burkholderia glumae* is a bacterial rice pathogen that can cause a fast, localized cell death in non-host *N. benthamiana.* We also cloned a green fluorescent protein gene (*GFP*) into the vector as a negative control, and HR symptoms were monitored within 3 days after agroinfiltration. Screening results 3 out of 20 candidate effectors had strong HR suppression ability, including *MiISE2* (Minc04520), *MiISE10* (Minc08615), and *MiISE6* (Minc06775) (**Figure 1**).

### Subcellular Localization of Three Candidate Effectors

**FIGURE 2:**
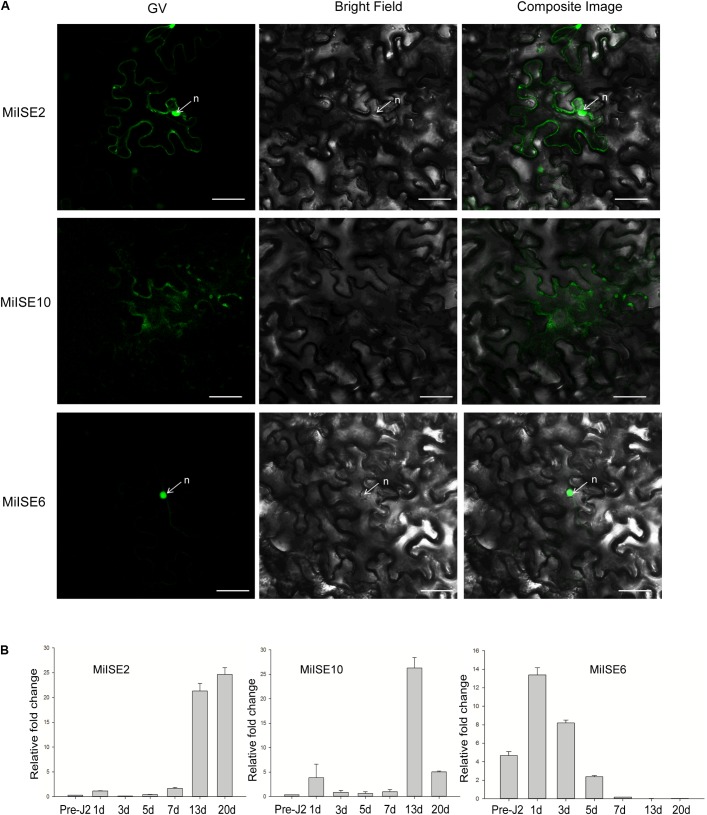
**(A)** Subcellular localization of C-terminally GFP-tagged candidate NLS effectors in *N. benthamiana* leaves. *N. benthamiana* leaves were monitored using confocal microscopy 24–48 h after transfection with effector::GFP construct. **(B)** The developmental expression of these effectors. The relative expression of effectors was quantified using q-PCR in four different *M. incognita* life stages: pre-J2s, par-J2s (1–3 days), J3s (7–13 days), and J4s (20 days). Each bar represents the mean plus SD of three biological replicates; with four technical replicates for each independent experiment. The 18S gene was used as an internal control. n, nucleus. Scale bars = 10 μm.

To investigate the subcellular localization of the three candidate effectors in plant cells, the green fluorescent protein (*GFP*) gene was fused with the effector (without signal peptide and stop codon) at its C terminus with the 35 S promoter, and the effector proteins were then transiently expressed in *N. benthamiana*. Confocal microscopy revealed distinct subcellular localization of the 3 candidate effectors. *MiISE2* shows nuclear-cytoplasmic localization; *MiISE10* appears to localize in the cytoplasm; and *MiISE6* is enriched in the nucleus (**Figure 2A**). The subcellular localization of *MiISE2* and *MiISE6* are consistent with the predictions by NucPred. Moreover, we used q-PCR to quantify the expression levels of three effectors during pre-parasitic J2 (pre-J2), parasitic J2 (par-J2), J3 and Female stages. The candidate effectors *MiISE2* and *MiISE10* have a high expression level in late parasitism stages (J3 and Female), but they exhibit a very low expression level in early parasitism stages. Conversely, the candidate effector *MiISE6* is upregulated in early sedentary stages, reaching a peak in the early par-J2 at 1 dpi, and then undergoing a dramatic reduction in the J3 parasitism stage (**Figure 2B**). The expression pattern of *MiISE6* is similar to other published nematode effectors which play central roles in nematode parasitism (Chronis et al., 2013; Lin et al., 2016; Chen et al., 2017), so we focus on *MiISE6* and speculate it may play an important role during the initial establishment of the plant-nematode interaction.

### Sequence and Homolog Analysis of *MiISE6* From *M. incognita*

**FIGURE 3:**
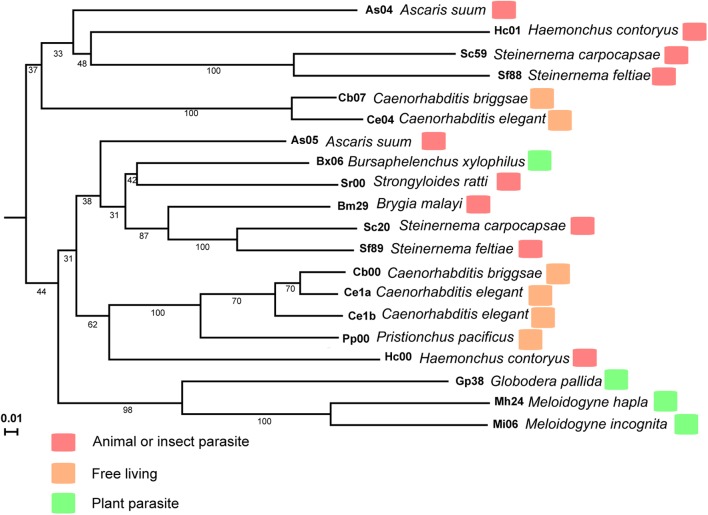
The phylogenetic tree of MiISE6 homologues inferred using the maximum likelihood method. The bootstrap value is given on each node.

The *M. incognita MiISE6* cDNA encodes a 158 amino acid protein with a predicted N-terminal signal peptide (SP, residues 1–32) for secretion; an OGFr_N region (residues 35–62); and two NLS motifs (residues 89–98, 109–118) (Supplementary Figure S2). A search of homologues of *MiISE6* from 16 nematodes species (including 4 free living nematodes, 6 animal or insect parasitic nematodes, and 6 plant parasitic nematodes) (Supplementary Table S1) was performed with BlastP (E ≤ 1e-10), and we obtained 20 homologues (Supplementary Table S3). These homologs were not only distributed in plant parasitic nematodes, but also in animal or insect parasitic nematodes and free-living nematodes. Based on the amino acid sequences of *MiISE6*, a phylogenetic tree was constructed in MEGA5 using the maximum likelihood method with 1000 bootstrap replicates. *MiISE6* was closest to the homologue from *M. hapla*, and they clustered with *MiISE6* homologues from *Globodera pallida* (**Figure 3**).

### Identification of Nuclear Localization Domains in MiISE6

**FIGURE 4:**
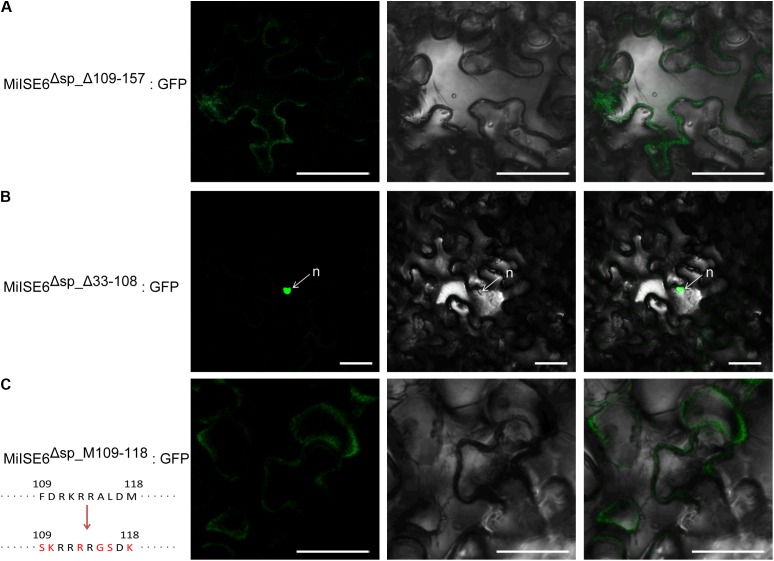
Identification of nuclear localization domains in MiISE6. C-terminally GFP-tagged MiISE6^Δsp_Δ33_108^, MiISE6^Δsp_MΔ109_118^, and MiISE6^Δsp_MΔ109_118^ were transiently expressed in *N. benthamiana* leaves, respectively. *N. benthamiana* leaves were monitored using confocal microscopy 24–48 h after transfection. **(A)** Cytoplasmic localization of MiISE6^Δsp_Δ109_157^:GFP. **(B)** Nuclear localization of MiISE6^Δsp_Δ33_108^:GFP. **(C)** Cytoplasmic localization of MiISE6^Δsp_MΔ109_118^:GFP. There was 5 amino acid substitution (red colored) in the NLS^109-118^ of MiISE6^Δsp_MΔ109_118^compared to MiISE6^Δsp^. n, nucleus. Scale bars = 10 μm.

Sequence analysis revealed that MiISE6 harbors two putative NLSs including ^89^GMQKRKRALD^98^ and ^109^FDRKRRALDM^118^. To test which NLS play function in nuclear localization, we firstly generate two constructs MiISE6^Δsp_Δ109_157^:GFP and MiISE6^Δsp_Δ33_108^:GFP, which contain NLS^89-98^ and NLS^109-118^, respectively. As shown in **Figure 4**, the MiISE6^Δsp_Δ109_157^:GFP localized to the cytoplasm (**Figure 4A**), whereas MiISE6^Δsp_Δ33_108^:GFP localized to the nucleus in *N. benthamiana* leaves (**Figure 4B**). The results indicate that the predicted NLS^89-98^ motif may not responsible for the nuclear localization of *MiISE6*. To determine whether NLS^109-118^ motif play role in nuclear localization, we used site-directed mutagenesis to generate MiISE6^Δsp_MΔ109_118^:GFP, and the fluorescence accumulation was observed in cytoplasm, and no fluorescence was observed in the nucleus (**Figure 4C**). These results indicate that the NLS^109-118^ lead to the nuclear localization of MiISE6.

### *MiISE6* Is Expressed in Subventral Esophageal Gland Cells

**FIGURE 5:**
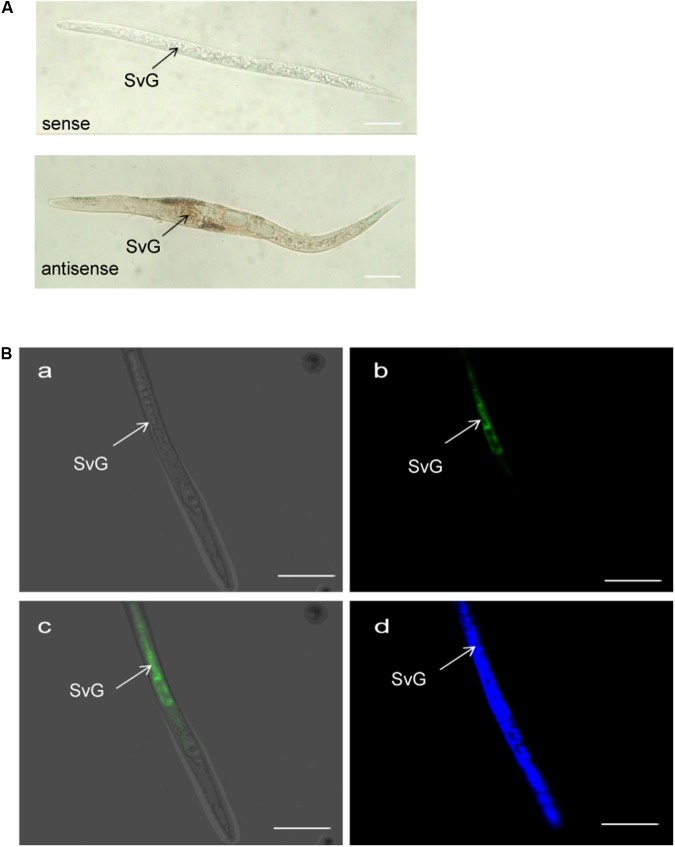
Spatial expression of *MiISE6*. **(A)** Digoxigenin-labeled *in situ* mRNA hybridization. **(B)** FITC-labeled *in situ* mRNA hybridization. Both digoxigenin-labeled antisense *MiISE6* cDNA probe and 5′ end labeled with FITC cDNA probe localized *MiISE6* transcripts within the subventral gland cells. **(B,a)** Bright Field. **(B,b)** GFP channel. **(B,c)** Composite Image. **(B,d)** DAPI channel.

The tissue localization of *MiISE6* transcripts in the nematode was verified using *in situ* hybridization. With digoxigenin-labeled antisense cDNA probes of *MiISE6*, there is a strong signal within the subventral gland cells of the pre-parasitic J2s. No signals are detected using the sense cDNA probes. Consistent with the result of DIG-labeled *in situ* mRNA hybridization, strong fluorescent signals are observed in the subventral esophageal gland by fluorescence *in situ* hybridization, and there is no tissue specificity when dyed with DAPI (**Figure 5**).

### *MiISE6* Contains Functional Signal Peptide

**FIGURE 6:**
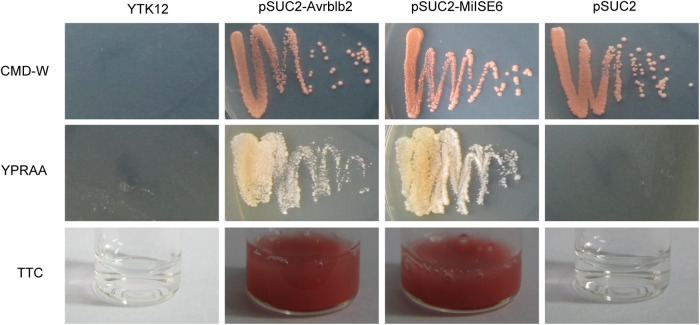
Functional validation of the signal peptides of MiISE6. Functional validation of the signal peptide of MiISE6 was performed using the yeast invertase secretion assays. Yeast YTK12 strain carrying the Avrblb2 and MiISE6 signal peptide, which expresses two signal peptide fragments fused in frame to the mature invertase gene SUC2, is able to grow in both the CMD-W media and YPRAA media (with raffinose instead of sucrose), as well as react with TTC and turn into red formazan, indicating secretion of invertase. YTK12 carrying the pSUC2 vector was used as negative control.

We used a yeast system requiring invertase secretion to grow on raffinose media to validate the function of the signal peptide of MiISE6. The predicted signal peptide sequence and the subsequent 24 amino acids of *MiISE6* was firstly fused with the SUC2 minus the signal peptide and then inserted in vector pSUC2T7M13ORI (Jacobs et al., 1997). The final construct enables the successful secretion of invertase, and then the invertase mutant yeast strain YTK12 can hydrolyze raffinose and grow on YPRAA media. The invertase secretion was confirmed with 2,3,5-triphenyltetrazolium chloride (TTC), which reacts with monosaccharides to form the insoluble (red colored) triphenylformazan. Compared with the negative control, the fusion of signal peptide of *Avrblb2* and *MiISE6* to the SUC2 enabled the secretion of invertase, which lead to the yeast cells growing on YPRAA media (**Figure 6**). These results revealed that the MiISE6 protein habor a functional signal peptide.

### *MiISE6* Increases *M. incognita* Parasitism

**FIGURE 7:**
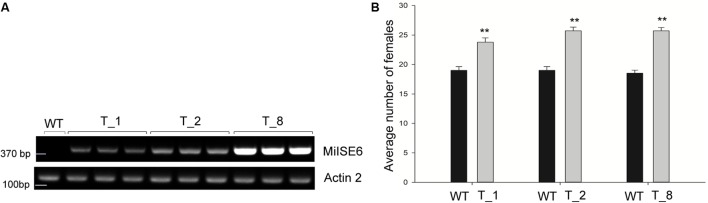
The overexpression of *MiISE6* in *Arabidopsis* promotes nematode susceptibility. **(A)** RT-PCR assays were used to determine the expression of *MiISE6* in three independent transgenic lines with Actin2 as reference. Upper image, agarose gel image of MiISE6; Lower image, agarose gel image of Actin2. **(B)** There was an increase of females in the T_1, T_2, and T_8 transgenic lines compared with WT Arabidopsis (Col-0). Independent samples *t*-tests were used for significance test between treatments, with a 95% confidence interval. Error bars represent standard Error (SED), and the mean values significantly different from the control are demoted by ^∗∗^ (^∗∗^*P* < 0.01). The experiments were performed with three independent replicates.

**FIGURE 8:**
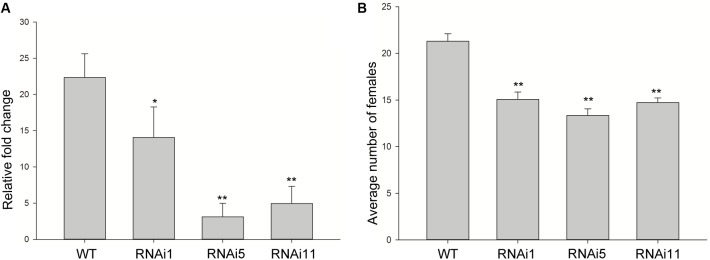
Host-derived *MiISE6* silencing effect on nematode parasitism. **(A)** q-PCR assays were used to determine the expression level of *MiISE6* in WT and three independent RNAi lines. **(B)** There was a reduction of females in three RNAi lines (RNAi1, RNAi5, RNAi11) compared with control plants. Independent samples t-tests were used for significance test between treatments, with a 95% confidence interval. Error bars represent standard Error (SED), and the mean values significantly different from the control are demoted by ^∗^ (^∗^*P* < 0.05, ^∗∗^*P* < 0.01). The experiments were performed with three independent replicates.

To determine whether the expression of *MiISE6* in *Arabidopsis* can change plant phenotype and nematode susceptibility, we generated transgenic *Arabidopsis* lines expressing ^Δsp^MiISE6 driven by the CaMV35S promoter. Three independent transgenic lines (T_1, T_2, T_8) were selected for phenotype observation and the nematode inoculation. We firstly detected the expression of transcripts in independent transgenic lines by RT-PCR. The photo (**Figure 7A** and Supplementary Figure S3) shows that *MiISE6* gene is really expressed in *Arabidopsis* plants. Then, we counted the number of root-knots in each transgenic plant, and compared the difference between each transgenic line and the wild-type plants by statistical significance test. The results show that there are more root-knots in each transgenic line plants than those in wild-type plants (mean 23–25 vs. 18–19) (Supplementary Figure S4). The difference is significant at a statistic level (all of the three tests with *P* < 0.001, Supplementary Figure S5). It indicates that all three transgenic lines are more susceptible to *M. incognita* than control, and there is an increase in females with an average rate of 25.15, 35.26, and 38.80%, respectively (**Figure 7B**). To further assess the role of *MiISE6* in nematode parasitism, we used a host-derived gene silencing method to knock down the expression of *MiISE6* in *M. incognita* during the nematode infection. Three RNAi lines (RNAi1, RNAi5, RNAi11), with at least 15 plants for each treatment, and three independent experiments. By RT-qPCR assays, it is shown that the expression of *MiISE6* in *M. incognita* collected from inoculated on RNAi plants at 3 dpi is significant reduced compared with those from the wild-type (WT) plants (Supplementary Figure S6 and **Figure 8A**). We also counted the number of root-knots in each RNAi plant and wild-type plant. Statistical analysis showed that there is an obvious reduction on RNAi plants (mean 13-15 knots) (Supplementary Figure S7), compared to those on wild type plants (mean 21). There was a 29.16, 35.26, and 30.86% reduction of females in three RNAi plants, respectively (**Figure 8B**). The results of *t*-tests show that the difference is significant (*P* < 0.001, Supplementary Figure S8). These data indicate that *MiISE6* is an important effector in mediating parasitic success of *M. incognita*.

### Identification of Differentially Expressed Genes (DEGs) Between MiISE6 Transgenic Plants and WT Plants

The difference in expression level of DEGs between WT and *MiISE6* transgenic *Arabidopsis* plants was calculated according to the FPKM method. Under the criteria of |log_2_FC|≥ 1 and FDR ≤ 0.01, a total of 852 genes were identified in *MiISE6*-overexpressing samples compared with WT samples, of which 772 were upregulated and 80 were downregulated. Detailed information is provided in Supplementary Datasheets S3, S4.

### GO and KEGG Enrichment Analysis of the DEGs

**FIGURE 9:**
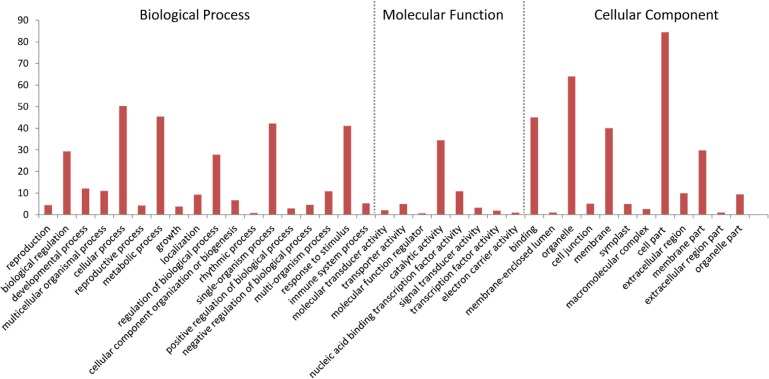
Gene ontology (GO) distribution of DEGs. All of the DEGs were assigned to three categories: cellular component, molecular function, and biological process.

**FIGURE 10:**
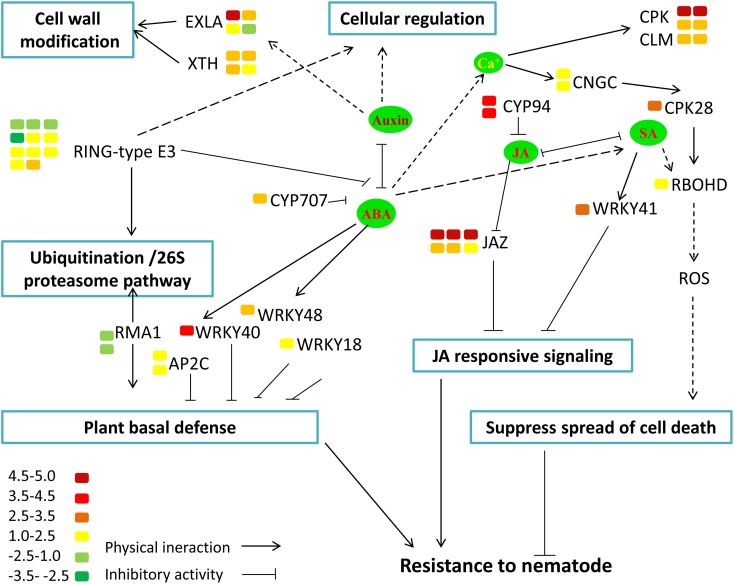
Model illustrating the interplay between potential DEGs involved in host defense responses. The color bar represents the expression level (log_2_FC) and number of DEGs.

To functionally classify the DEGs, gene ontology (GO) terms were assigned to each DEG using AgriGO based on the TAIR 10 database. DEGs are categorized into three groups: Biological Process (BP), Cellular Component (CC), and Molecular Function (MF). The majority of the DEGs are assigned to the BP category, such as cellular process, metabolic process, and response to stimulus. In addition, we find that DEGs are significantly involved in binding, catalytic activity, and transcription regulator activity in the MF category. The results also show that in the CC category, the majority of the DEGs are significantly related with cell part and organelle (**Figure 9**). The expression of *MiISE6* in *Arabidopsis* leads to changes in multiple plant immune responses, including hormones signaling pathway, ubiquitination proteasome pathway, and calcium signaling pathway (Supplementary Table S4). In “regulation of defense response (GO:0031347)”, most enriched genes work as negative regulator of plant defense responses, such as WRKY40, WRKY18, and NUDT7(Yang et al., 2006; Ge et al., 2007; Pandey et al., 2010). In “protein ubiquitination (GO:0016567),” most enriched genes are plant U-BOX E3 ubiquitin ligases (PUBs). The U-Box domain mediates the binding to the conjugating enzyme (E2) during ubiquitination process. Previous study showed that there was a reduced bacteria infection in *pub22*/*pub24* double mutant and *pub22*/*pub23*/*pub24* triple mutant compared to control. Although the molecular mechanism is not well clarified, previous studies indicated that those genes may act as negative regulator of PAMP-triggered immunity by altering the activity of 26S proteasome (Stegmann et al., 2012; Cho et al., 2015). In “response to hormone stimulus,” genes involved in modulating multiple hormone signaling pathway, including JA signaling (GO:0009867), SA signaling (GO:0009751) and auxin signaling (GO:0009733). Moreover, we find that a number of genes are enriched in “cell wall modification (GO:0042545),” such as xyloglucan endotransglucosylase-hydrolase (XTHs) and expansin-like gene (EXLA). Those genes involved in the reorganization or loosening of cell walls (Cosgrove, 2000; Olsen and Krause, 2017). Recent studies have suggested that diverse pathogen acquired and exploited these plant derived enzymes for successful parasitism (Balestrini et al., 2005; Nikolaidis et al., 2014; Olsen and Krause, 2017). Our result shows that most of those genes exhibit a significant higher expression level in the *MiISE6* transgenic plants compared to control. Based on the present data, we draw a model illustrating the interplay between potential DEGs involved in host defense responses (**Figure 10**).

**FIGURE 11:**
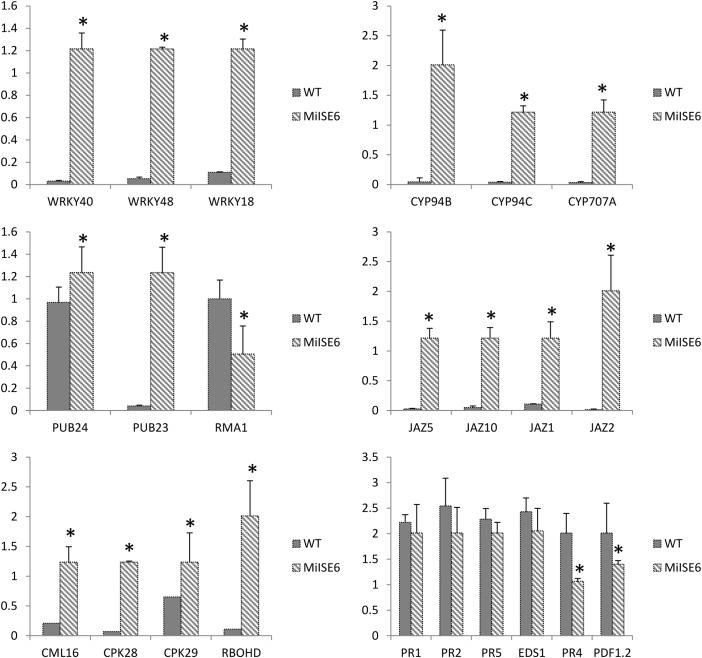
Verification of gene expression patterns in WT and *MiISE6* transgenic *Arabidopsis*. 23 DEGs were selected to validate the reliability of the RNA-seq results. Actin2 (AT3G18780) was used as an internal control to normalize the expression of the genes in each corresponding q-PCR samples. Each bar represents the mean plus SD of three biological replicates, with four technical replicates for each independent experiment, and the mean values significantly different from the control are demoted by ^∗^ as determined by independent samples *t*-test (*P* < 0.05).

Moreover, the results of KEGG enrichment analysis indicate that a total of 228 annotated DEGs are assigned into 25 pathways. As shown in Supplementary Table S5, these pathways are mainly classified into 8 categories (*P* ≤ 0.05). The most significantly enriched KEGG pathway is “Plant–pathogen interaction,” and most genes enriched in this pathway are calmodulin-related proteins or calcium dependent kinases, such as CML16, CPK29, CPK28. The expression of CPK28 has a 5.3-fold increase (log_2_FC = 2.31) in the *MiISE6* transgenic plants compared to the WT plants. Previous studies have proven that CPK28 can interact with phosphorylate BIK, and contribute to BIK1 turnover (Monaghan et al., 2014, 2015); the overexpression of CPK28 in *Arabidopsis* inhibits PTI signaling and immunity (Matschi et al., 2015). Moreover, the NADP oxidase RBOHD is also enriched in the “Plant–pathogen interaction” pathway and presents a higher expression in the transgenic plants, and the expression of SA-responsive genes EDS1, PR1, PR2, and PR5 did not show significant differences in the transgenic plants compared to control. Additionally, in the KEGG pathway “Plant hormone signal transduction,” most enriched genes are JAZ genes, which work as repressors of jasmonate signaling (Chini et al., 2007), and they all showed significantly higher expression in the transgenic plants compared to control. Conversely, the JA-responsive genes PR4 and PDF1.2 showed a significant decrease in the transgenic plants. The expression patterns of these genes were verified by q-PCR, and the results are similar to the transcriptome analysis (**Figure 11**).

## Discussion

Studies on identification of *Meloidogyne* spp. pathogenicity-related effectors have been conducted since *Meloidogyne* genome sequences were first reported. Most of these effectors are synthesized in the esophageal gland and subsequently delivered into plant cells and tissues through the stylet, including cell wall modifying enzymes (Smant et al., 1998; Bera-Maillet et al., 2000), antioxidant enzymes, and some enzymes that function in suppressing host defenses and manipulating plant signaling pathways (Doyle and Lambert, 2003; Dubreuil et al., 2011). Previous studies revealed that *Meloidogyne* effectors may target different subcellular compartments to counteract plant defenses, and a significant number of these effectors are specifically localized to the plant cell nucleus, such as 7H08, Msp40 and MgGPP (Zhang et al., 2015; Niu et al., 2016; Chen et al., 2017). Increasing evidence suggests that nuclear localized pathogen effectors play a central role in parasitism, by co-opting the host cell nuclear transport system and hijacking the host transcriptional machinery to subvert plant immunity (Kalderon et al., 1984a,b; Garcia-Rodriguez et al., 2006; Kay et al., 2007; Weinthal et al., 2011). In order to screen for more NLS effectors, in this study, based on RNA-seq and bioinformatics analysis, we predicted 110 putative secreted NLS effectors. From 20 randomly selected candidates, we identified three of them (MiISE2, MiISE10, and MiISE6) have strong HR suppression ability by utilizing a pEDV-based type III secretion system. We found the three candidates have distinct subcellular localization in *N. benthamiana*, and distinct expression in different stages of the nematode. We finally selected MiISE6 for function verification, because it can target the host nucleus, and is predominantly upregulated in the parasitic J2 stage. Our results confirmed MiISE6 as an effector involved in plant-nematode interaction, which can enhance the nematode parasitism by interfering multiple signaling pathways of plants, especially suppression of JA signaling pathway. The other two (MiISE2, MiISE10) are waiting for functional verification. Moreover, in this study, only 20 candidates were randomly selected for functional assay, and a large part of predicted NLS effector candidates are under exploring. We believe more effectors in *M. incognita* will be identified in the future. Furthermore, transcriptome datasets in our study may provide useful information for further study in nematode–plant interactions.

Sequence analysis of MiISE6 showed that it contains a signal peptide, an OGFr_N domain and two NLS motifs. We have determined the secreted activity of SP and nuclear localization of NLS motif. About OGFr_N domain, it is found in the opioid growth factor receptor (OGFr), which is an integral membrane protein associated with the nucleus and plays important roles in the regulation of cell growth (Zagon et al., 2002). OGFrs are widely reported in the phylum in human, rat and mouse cells (Zagon et al., 1994, 1999; Kren et al., 2015). It was reported that the OGFr was initially expressed on the outer nuclear envelope, and can transport into the nucleus through the nuclear pore (Zagon et al., 2003, 2005; Cheng et al., 2010). Previous studies showed that in sequence of OGFr there is one or more NLS accompanied with OGFr_N domain, which respond for the nuclear transporting of OGFr genes (Cheng et al., 2009). Based on the conserved domain analysis, we found that MiISE6 was a putative OGFr gene, containing an OGFr_N domain and subsequent two NLS motifs, and one motif NLS^109-118^ is confirmed to work for entering nuclei of plant cells by using sequence deletion variants and site-directed mutagenesis. Sequence analysis showed that identity of protein sequences between MiISE6 and homologs of *M. hapla* and *Globodera pallida* are more than 68 and 46%, respectively. We suppose that similar efforts of MiISE6 may also exist in these two nematodes, perhaps it is a common parasitism mechanism in sedentary PPNs.

Sedentary PPNs establish long term relationships with their hosts. Based on the current studies and previous literature, mechanisms of these effectors in parasitism has been elucidated that PPNs can manipulate plant hormone to facilitate successful invasion or establish feeding cells. When plants were invaded by PPN, plant hormones have pivotal roles in the regulation of immune responses toward attack. Nematode induce feeding cells by manipulating many aspects of plant development, which involve auxin transport and plant cell differentiation pathways. It was reported that the effector Hs19C07 of *H. schachtii* can interact with an auxin influx transporter LAX3 of plant, and the overexpression of this gene in *Arabidopsis* increased lateral root emergence, indicating a modulating of auxin influx into root cells (Lee et al., 2011). Another *H. schachtii* effector 10A07 can directly target IAA16 to manipulate auxin responses. Remarkably, the 10A07 transgenic plants showed hypersusceptibility to nematode infection (Hewezi et al., 2015). Auxin is known to involve in cell expansion and cell wall breakdown, accompanied by changes in the expression of plant cell wall modifying proteins (Szakasits et al., 2009), which is important for development of feeding structure. In this study, we found that the expression of some expansin-like genes and xyloglucan endotransglucosylase-hydrolases are obviously upregulated in transgenic *Arabidopsis*. Expansions are proposed to breakdown the adjacent between xyloglucans and cellulose, resulting in plant cell wall loosing (Cosgrove, 2000). Similar to expansin-like genes, xyloglucan endotransglucosylase-hydrolases also work in loosing cell walls by catalyzing the cleavage and rejoining of the xyloglucans to the primary cell wall (Van Sandt et al., 2007). In addition, the overexpression of XTHs in plants is compatible with a potential role in xyloglucan degradation, which contributing to wall extension (Baumann et al., 2007). We speculate that the expression of *MiISE6* in *Arabidopsis* may accelerate the breakdown of cell wall through interfering the auxin pathway, which help nematode migrating and giant cell formation in plant roots.

Previous studies have identified the signaling molecules jasmonic acid (JA) and salicylic acid (SA) as important players in induced defense of the plant against pathogens, and the JA and SA signaling can be exploited by pathogens to facilitate parasitism (Gutjahr and Paszkowski, 2009). It was found that a *H. glycines* effector 10A06 can stimulate polyamine biosynthesis by interacting with Spermidine Synthase 2 (SPDS2), which lead to the disruption of SA-mediated defense signaling (Hewezi et al., 2010). A general view is that SA plays an important role in defense against biotrophic pathogens and JA play role in defense against necrotrophs (Glazebrook et al., 2003). Recent studies have illuminated that JA may play a more dominant role in the plant-pathogen interactions in roots (Fujimoto et al., 2011; Nahar et al., 2011; Mendy et al., 2017). Based on the data from this study, we found that the expression of *MiISE6* in *Arabidopsis* resulted in the upregulation of JAZ genes, which are known as repressors of JA signaling (Chini et al., 2007; Thines et al., 2007; Demianski et al., 2012). It was reported that eight of twelve JAZ genes were induced after DC3000 infection, and the disruption of JAZ10 resulted in the increased susceptibility of *Arabidopsis* to DC3000 (Demianski et al., 2012). It was hypothesized that JAZ genes attenuate JA signaling by negative feedback regulating the degrading of JAZ proteins by SCF^COL1^ (Chico et al., 2008). Moreover, the JA defense response marker genes PR4 and PDF1.2 are downregulated in the transgenic plants, those indicated that the expression of *MiISE6* in *Arabidopsis* can suppress the JA signaling. In addition, we also found that the expression of *MiISE6* in *Arabidopsis* activated the expression of CPK28 and then resulted in the accumulation of RBOHD(Matschi et al., 2015); however, the expression of SA-responsive genes *PR1*, *PR2*, *PR5*, and *EDS1* did not show detectable changes in the transgenic plants compared to control. A recent study has demonstrated that parasitic nematodes can restrict infected cell death and promote nurse cell formation by activating the expression of NADPH oxidase RBOHD to produce ROIs (Siddique et al., 2014). As in the transgenic plants, the SA-mediated defense responses were not activated, so we speculated that the concentration of ROIs may be at a low level and may function as signaling molecules to suppress HRs and maintain the development of the feeding site.

All aspects of plant physiology and development are controlled by ubiquitin-proteasome pathway, which function in regulating synthesis of new polypeptides and degradation of existion proteins (Craig et al., 2009). Previous studies have revealed that the ubiquitination system can be exploited by plant pathogens to suppress host immune defense responses (Birch et al., 2009; Dielen et al., 2010). It is reported that a *Globodera rostochiensis* effector GrUBCEP12 can cleave into free ubiquitin and a CEP12 peptide *in planta*, which play important role in host immunity suppression and affecting the host 26S proteasome to promote parasitism. In this study, we found that the overexpression of MiISE6 in *Arabidopsis* lead to the upregulation of PUBs, which act as negative regulator of PAMP-triggered immunity by altering the activity of 26S proteasome, that indicates MiISE6 may also interfering the ubiquitination proteasome pathway to facilitate parasitism.

## Conclusion

The overexpression of *MiISE6* in host interferes multiple signaling pathways. As different plant signaling may function as networks in host defense responses (Berens et al., 2017), further work such as the target of MiISE6 are required to elucidate the role of MiISE6 during plant-nematode interaction.

## Author Contributions

QS and ZM performed the experiments, transcriptome analysis and manuscript. XpZ and XZ did the quantitative real-time PCR. JL and RLin contributed the phylogenetic analysis. RLiu completed the nematode inoculation. YW and YY contributed to the manuscript revision. BX and XC contributed to the study design. All authors read and approved the final manuscript.

## Conflict of Interest Statement

The authors declare that the research was conducted in the absence of any commercial or financial relationships that could be construed as a potential conflict of interest.

## Footnotes

1 http://www.cbs.dtu.dk/services/SignalP/

2 http://www.predisi.de/

3 http://phobius.binf.ku.dk/

4 http://www.cbs.dtu.dk/services/TargetP/

5 http://www.cbs.dtu.dk/services/TMHMM-2.0/

6 http://systemsbiology.cau.edu.cn/agriGOv2/
