# Supplementary material for: The Novel Secreted Meloidogyne incognita Effector MiISE6 Targets the Host Nucleus and Facilitates Parasitism in Arabidopsis
Source: Front Plant Sci. 2018 Mar 23;9:252. doi: 10.3389/fpls.2018.00252 (PMC5876317; doi:10.3389/fpls.2018.00252)

**Supplemental information**

**The novel secreted *Meloidogyne incognita* effector MiISE6 targets the host nucleus and facilitates parasitism in *Arabidopsis***

Qianqian Shi^1,2^ ^†^, Zhenchuan Mao^1^ ^†^, Xiaoping Zhang^3^, Jian Ling^1^, Runmao Lin^1,4^, Xi Zhang^1,4^, Rui Liu^1^, Yunsheng Wang^1^, Yuhong Yang^1^, Xinyue Cheng^4^* and Bingyan Xie^1^*

^1^*Institute of Vegetables and Flowers, Chinese Academy of Agricultural Sciences, Beijing 100081, China*

^2^*Department of Plant Pathology and the Ministry of Agriculture Key Laboratory for Plant Pathology, China Agricultural University, Beijing 100193, China*

^3^*School of medical science, Chifeng University, Chifeng 024000, China*

^4^*College of Life Science,* *Beijing Normal University, Beijing 100875, China*

*Corresponding Email: [xiebingyan@caas.cn](mailto:xiebingyan@caas.cn); chengxy@bnu.edu.cn

**Supplemental Table S1. The platforms about** **nematodes genome and transcripts used in the homologue analysis in this study.**

| Species | Blast server links |
| --- | --- |
| *Meloidogyne incognta* | https://www.ncbi.nlm.nih.gov/genome/?term=meloidogyne+incognita |
| *Meloidogyne hapla* | https://www.ncbi.nlm.nih.gov/genome/?term=meloidogyne+hapla |
| *Meloidogyne floridensis* | https://www.ncbi.nlm.nih.gov/genome/?term=meloidogyne+floridensis |
| *Globodera pallida* | https://www.ncbi.nlm.nih.gov/genome/?term=Globodera+pallida |
| *Globodera rostochiensis* | https://www.ncbi.nlm.nih.gov/genome/?term=Globodera+rostochiensis |
| *Bursaphelenchus xylophilus*  *Ascaris suum*  *Brugia malayi*  *Haemonchus contortus*  *Steinernema carpocapsae*  *Steinernema feltiae*  *Strongyloides ratti*  *Caenorhabditis briggsae*  *Caenorhabditis elegans*  *Caenorhabditis remanei*  *Pristionchus pacificus* | https://www.ncbi.nlm.nih.gov/genome/?term=Bursaphelenchus+xylophilus  https://www.ncbi.nlm.nih.gov/genome/?term=ascaris+suum  https://www.ncbi.nlm.nih.gov/genome/?term=brugia+malayi  https://www.ncbi.nlm.nih.gov/genome/?term=heamonchus+contortus  https://www.ncbi.nlm.nih.gov/genome/?term=steinernema+carpocapsae  https://www.ncbi.nlm.nih.gov/genome/?term=steinernema+feltiae  https://www.ncbi.nlm.nih.gov/genome/3496  https://www.ncbi.nlm.nih.gov/genome/40  https://www.ncbi.nlm.nih.gov/genome/41  https://www.ncbi.nlm.nih.gov/genome/253  https://www.ncbi.nlm.nih.gov/genome/?term=pristionchus+pacificus |

**Supplemental Table S2. Primers used in this study (The lowercase regions represent the fusion sequence, the underlined regions represent cleavages sites of restriction enzymes).**

| Gene ID | Primers | Sequence | | Usage |
| --- | --- | --- | --- | --- |
| GPF | pEDV_GFP_F | ACGCGTCGACATGGTGAGCAAGGGCGAG | |  |
|  | pEDV_GFP_R | CGCGGATCCCTTGTACAGCTCGTCCATGCC | |
| Minc10706 | pEDV_MiISE1_F | ACGCGTCGACATGAATTTACAATATCACGAACACC | |  |
|  | pEDV_MiISE1_R | CGCGGATCCATCAGCAATCGCTGTTTCATC | |  |
| Minc04520 | pEDV_MiISE2_F | ACGCGTCGACATGTATGATTTATCCTCAGTTTTGG | |  |
|  | pEDV_MiISE2_R | CGCGGATCCTGCCTTATTTTTGTCTTCTAGTCC | |  |
| Minc02929 | pEDV_MiISE3_F | ACGCGTCGACATGGAACAGAACGTAAACAAC | |  |
|  | pEDV_MiISE3_R | CGCGGATCCTGTACAAGTTCCATTCTCATTT | |  |
| Minc01465 | pEDV_MiISE4_F | ACGCGTCGACATGAATCAAAATCCTTGTCATC | |  |
|  | pEDV_MiISE4_R | CGCGGATCCGTGTCGAGATCTTGATGCTG | |  |
| Minc06775 | pEDV_MiISE6_F | ACGCGTCGACATGAGCAACAACAACAATATTAACA |  | |
|  | pEDV_MiISE6_R | CGCGGATCC CATTAATAGCGCCCTTTTTTG | |  |
| Minc02099 | pEDV_MiISE7_F | ACGCGTCGAC ATGGACTGTCCTGCAGGTTG | |  |
|  | pEDV_MiISE7_R | CGCGGATCCTGGAGAGCATGGACGTTTG | |  |
| Minc01683 | pEDV_MiISE8_F | ACGCGTCGACATGTATGATTTATCCTCAGTTTTG | |  |
|  | pEDV_MiISE8_R | CGCGGATCCATTGACCATTTTGCAACC | |  |
| Minc08615 | pEDV_MiISE10_F | ACGCGTCGACATGGCCGAACTAGTAGAATTTCTCT | |  |
|  | pEDV_MiISE10_R | CGCGGATCCACTTAATTTTCTTTTTATTCTATTTTCCAC | | Effector |
| Minc08774 | pEDV_MiISE11_F | ACGCGTCGACATGCAACAACAAAAACAGAGAG | | screening for HR |
|  | pEDV_MiISE11_R | CGCGGATCCATCATCATCAGCTCCACTATTAAC | | Suppression |
| Minc08636 | pEDV_MiISE12_F | ACGCGTCGACATGGTTAAACCAAAGATGG | |  |
|  | pEDV_MiISE12_R | CGCGGATCCTAATTTTAACTCCAAATCTATACTT | |  |
| Minc07350 | pEDV_MiISE14_F | ACGCGTCGACATGGAAGTCTCTCATTCAAATAAAG | |  |
|  | pEDV_MiISE14_R | CGCGGATCC CAATTCACTCTTCATTGAATCATT | |  |
| Minc02258 | pEDV_MiISE15_F | ACGCGTCGACATGACGCGCACTGAACG | |  |
|  | pEDV_MiISE15_R | CGCGGATCCATAATTCCCCTCAATAAAGAAATG | |  |
| Minc18745 | pEDV_MiISE17_F | ACGCGTCGACATGGCTAAACGTAAAAAATCC | |  |
|  | pEDV_MiISE17_R | CGCGGATCCATAATGTCCTCTTTGTCGTTGT | |  |
| Minc04469 | pEDV_MiISE18_F | ACGCGTCGACATGAACTCGGTTAAAGAACAAAT | |  |
|  | pEDV_MiISE18_R | CGCGGATCCTATTACATCAGAGCTCATTCCAT | |  |
| Minc05591 | pEDV_MiISE19_F | ACGCGTCGACATGCGCCAGTCAATAACTT | |  |
|  | pEDV_MiISE19_R | CGCGGATCCCATTTTTAACATCCCAAACAT | |  |
| Minc11561 | pEDV_MiISE20_F | ACGCGTCGACATGTGGGGAGATGATTTCTTC | |  |
|  | pEDV_MiISE20_R | CGCGGATCCGCAGTTAGAATATTTCTTTACAACC | |  |
| Minc00196 | pEDV_MiISE21_F | ACGCGTCGACATGGTTAAAAATAATAAAAATC | |  |
|  | pEDV_MiISE21_R | CGCGGATCCAAAATTCGGATCGTCC | |  |
| Minc13882 | pEDV_MiISE22_F | ACGCGTCGACATGATCAATTCACTCACTTCTCG | |  |
|  | pEDV_MiISE22_R | CGCGGATCCTTCTTCATCTTCCAAATCCG | |  |
|  | DIG-MiISE6-F | ATGAGCAACAACAACAATATTAACA | | *In situ* |
|  | DIG-MiISE6-R | CATTAATAGCGCCCTTTTTTG | | hybridization |
|  | pSUC2_MiISE6_F | CCGGAATTCATGACTTATTTAACAGAAAATTTTG | | SP verification |
|  | pSUC2_MiISE6_R | CCGCTCGAGTTCTGTTCCAACAAGTATAGGTG | |  |
|  | PCB_MiISE2_F | CATGCCATGGCATGTATGATTTATCCTCAGTTTTGG | |  |
|  | PCB_MiISE2_R | CTAGACTAGTTGCCTTATTTTTGTCTTCTAGTCC | |  |
|  | PCB_MiISE10_F | CATGCCATGGCATGGCCGAACTAGTAGAATTTCTCT | | Subcellular |
|  | PCB_MiISE10_R | CTAGACTAGTACTTAATTTTCTTTTTATTCTATTTTCCAC | | localization |
|  | PCB_MiISE6_F | CATGCCATGGATGAGCAACAACAACAATATTAACA | |  |
|  | PCB_MiISE6_R | CTAGACTAGTCATTAATAGCGCCCTTTTTTG | |  |
|  | PCB_MiISE6^Δ109_157^_F | CATGCCATGGATGAGCAACAACAACAATATTAAC | |  |
|  | PCB_MiISE6^Δ109_157^_R | CTAGACTAGTACCCCCTCCAAACCCAT | |  |
|  | PCB_MiISE6^Δ33_108^_F | CATGCCATGGTTTGATAGAAAAAGAAGAGCTTTG | |  |
|  | PCB_MiISE6^Δ33_108^_R | CTAGACTAGTCATTAATAGCGCCCTTTTTTG | |  |
|  | PCB_MiISE6 ^MΔ109_118^_F1 | TCTGAGAGAAGAAGAAGAGGTTCGG | |  |
|  | PCB_MiISE6 ^MΔ109_118^_R1 | ACCTCCCCCAAGACTCTCTTCTTCT | | Site-directed |
|  | PCB_MiISE6 ^MΔ109_118^_F2 | AGAGGTTCGGATAAGATGGAAGGGG | | mutagenesis |
|  | PCB_MiISE6 ^MΔ109_118^_R2 | ATCTTTTTCTTCTCCAAGCCTATTC | |  |
|  | pEGAD_MiISE6_F | CCGGAATTCATGAGCAACAACAACAATATTAACA | | Arabidopsis |
|  | pEGAD_MiISE6_R | CGCGGATCCCATTAATAGCGCCCTTTTTTG | | overexpression |
|  | CHSA-F | CAATCGATGATTTAAATGTGTAAG | | Checking CHAS intron |
|  | CHSA-R | GACTAAAAAGGAAATATTGTCACAT | |  |
|  | pFGC_MiISE6_sense_F | CATGCCATGGATGAATGATTGTTTAATTGCC | | Host-derived RNAi |
|  | pFGC_MiISE6_sense_R | TTGGCGCGCCACTTAATTTTCTTTTTATTCTATTTTC | |  |
|  | pFGC_MiISE6_antisense_F | CTAGTCTAGAATGACTTATTTAACAGAAAATTTTGGAAT | |  |
|  | pFGC_MiISE6_antisense_R | CGCGGATCCTTACATTAATAGCGCCCTTTTTTG | |  |
|  | RT_MiISE6_F | ATGAGCAACAACAACAATATTAACA | | RT PCR |
|  | RT_MiISE6_R | CATTAATAGCGCCCTTTTTTG | |  |
|  | q_MiISE6_F | TTCAATTCCTTTCATCAACATCC | | q-PCR |
|  | q_MiISE6_R | TCTTTTCCTCTTCTGCATTCCAA | |  |
|  | q_MiISE2_F | CAGTCATTTACCTCCCCAAGAA | |  |
|  | q_MiISE2_R | ACAGTATCCCCAACATTAGCCC | |  |
|  | q_MiISE10_F | GCCCAATGGGTTCCCGTTCTA | |  |
|  | q_MiISE10_R | GACGCAGTCTTCGGGGAGTTT | |  |
| U81578 | 18S_F | ACCGTGGCCAGACAAACTAC | |  |
|  | 18S_R | GATCGCTAGTTGGCATCGTT | | q-PCR (control) |
| AT1G80840 | q_WRKY40_F | GTTATTGGCGGAGTGTCGGAG | | q-PCR |
|  | q_WRKY40_R | GGAGCACAAGCACATTTGAAGT | |  |
| AT5G49520 | q_WRKY48_F | TTCATTCGATGCCTTTCCTCTC | |  |
|  | q_WRKY48_R | TGGCTTAGTACCCTTTTGCTCTT | |  |
| AT4G31800 | q_WRKY18_F | GGAGGTCTCAGTTTTGGCTTCT | |  |
|  | q_WRKY18_R | CGGTATATCGGTCTGCTCGATT | |  |
| AT3G48520 | q_CYP94B_F | CTGAACCGGGGAGTACACGAC | |  |
|  | q_CYP94B_F | ACCTTAAGCCCACCAGCCATA | |  |
| AT2G27690 | q_CYP94C_F | GTCGGGATACGGTAGCTGCTG | |  |
|  | q_CYP94C_F | GCGAATTTGGAATCGAATTGA | |  |
| AT5G45340 | q_CYP707A_F | CTCCGGTTTGTTTCTCACTCTCT | |  |
|  | q_CYP707A_F | TCCGTATCTTCTCTGTTTTGCTG | |  |
| AT1G20823 | q_RING1_F | CTTGGTTGTTGCCAGGTGTCA | |  |
|  | q_RING1_R | CATCTTCGCCTTGCTTGATTC | |  |
| AT2G35930 | q_RING2_F | GAACCACGGAGCAGCCATAG | |  |
|  | q_RING2_R | ACCTTCCCACCGACAACAAAA | |  |
| AT4G03510 | q_RMA1_F | TCTGTGGTCACCTCTTTTGCTG | |  |
|  | q_RMA1_R | AACCGGTCCTACGGGTCTTTTA | |  |
| AT1G17380 | q_JAZ5_F | TTCCCTCCATCGATTCTTTGC | |  |
|  | q_JAZ5_R | CGGTCTTTGTGACGACTGTCCT | |  |
| AT5G13220 | q_JAZ10_F | CGTTCGGTTCCGTCTACTCC | |  |
|  | q_JAZ10_R | GCTGCTTCATTAGCGACCTTC | |  |
| AT1G19180 | q_JAZ1_F | TTTCTTCCTCATCTTCCTCTCTTCC | |  |
|  | q_JAZ1_F | GATTGGCTCTTGTGTTGTGGTTT | |  |
| AT1G74950 | q_JAZ2_F | ACGGTGGTCGAGTTATGGTGTT | |  |
|  | q_JAZ2_F | ATTGGCTCTTGTGCTGCGG | |  |
| AT3G25600 | q_CML16_F | CGCCGCTCTTCTGCGTTCTCT | |  |
|  | q_CML16_R | AGCTTCCGTCATCATTTCCGT | |  |
| AT5G66210 | q_CPK28_F | CAGTAGACGAAGCAGTCAGACCA | |  |
|  | q_CPK28_R | CTCAACAGCAATAGGAAGAACCA | |  |
| AT1G76040 | q_CPK29_F | ACCAACATAAAACGACCAAAAAC | |  |
|  | q_CPK29_R | CTTGAATCGGAAGAGGAAGAGAT | |  |
| AT5G47910 | q_RBOHD_F | CTGGACACGTAAGCTCAGGA | |  |
|  | q_RBOHD_R | GCCGAGACCTACGAGGAGTA | |  |
| AT2G14610 | q_PR1_F | AAGGAGCATCATATGCAGGA | |  |
|  | q_PR1_R | ATTTAAATAGATTCTCGTAATCTCAGC | |  |
| AT3G57260 | q_PR2_F | CTTGAACGTCTCGCCTCCAGTC | |  |
|  | q_PR2_R | TCCAGAAACCGCGTTCTCGATG | |  |
| AT1G75040 | q_PR5_F | CAATTGCCCTACCACCGTCTGG | |  |
|  | q_PR5_R | CTTAGACCGCCACAGTCTCCG | |  |
| AT3G48090 | q_EDS1_F | CTCTCTCTCTCTTTTCATACTGTGC | |  |
|  | q_EDS1_R | CTTCTGATTCTCTACTCGTTTCTTC | |  |
| AT3G04720 | q_PR4_F | GTACGCGCCACCTACCATTT | |  |
|  | q_PR4_R | GCATTTGTTCTTGTGTTCTTCACC | |  |
| AT5G44420 | q_PDF1.2_F | TTATCTTCGCTGCTCTTGTTCTCT | |  |
|  | q_PDF1.2_R | CATGATCCATGTTTGGCTCCT | |  |
| AT3G18780 | actin2-F | TTGACAGAGAAGAACAAGGAAGAA | | q-PCR /RT-PCR(control) |
|  | actin2-R | GGAAGAAGATGAGATTGAGGAAGA | |  |

**Supplementary Table 3. The corresponding Gene ID and amino acid sequence of the homologs of MiISE6 in the phylogenetic tree.**

| Gene name | Corresponding gene ID | Amino acid sequence |
| --- | --- | --- |
| As04 | ASU_04673 | MKTVSLPNRLLKACTGGRILRSKRAFDRLDESALGLFRKRSFDRIDGSAFGPHRHKKAFDRIDGSDFGLVKRAFDRIEGAGFGLSKRAFDRIEGSGFGLDKRAFDRLEGSDFGLVKRAFDRLEGSDFGLDKRAFDRVGESNFGFDKRSFDRVADSTAFRLSRGAFSRIDDNDFGLFKLNAVNPGTENTFTTETSFRSH |
| Hc01 | HCOI01334800.t1 | MTQYSSLLRLHLVQMVFLALCSAYPYVLNSDYELTDVAERPFGGLTKRAFDRIESNDFGLFKRSAAKRAFDRIEMADFGFRRKRAFDRVGRTEFGFEGVLRKRAADRLADIGFRNKRGLDQLDGTDLGLMFDPVRPAREELIDRLAYTIAAMGHATPVAISAVPLVDDQK |
| Sc59 | L596_g19643.t1 | MRTNNLGHVPLTLLSLTVTLAYSLPYAVVVGSNTAPSIVPSGHLTSKRAFDRLDMSPFDFGAYRKRAFDRLDESAFGFTRKRRAFDRLDESVFGLMAARRRRAFDRLEQSGFGLVKKRSFDRLDSGNFGFGMGKRSGNYVIPAHALAKRPFDRLERSPFGLSKRSQKIALGPEVVDLLGEFRPSAVDF |
| Sf88 | L889_g18954.t1 | MFPDSRDCVGPRRCAVDSPTVIRLEVKLDVGGALGDRANLDLHLNALKRSSPAVVQSRPNPSPTQFCDAINFDARISANPLLGASVPSERADRLFHSRVSSRLMQGFGEPFGARIWPASDQQTFAWSKESNFAAEKRRFVDGATKSEPFAKTHSLQNAIHRWIRSRSRKAPGMRANSLGHVLLALLSLTVTLAASLPYALVLSSPATSGHVTSKRAFDRLDMSPFDFGAYRKRAFDRLEQSDFGFMARKRRAFDRLDESVFGLMAARRRRAFDRIEQSGFGLVKKRAFDRLDSGNFGFGMGKRSVDYVIPSHSLSKRPFDRLERSAFGLNKRSQAIALGPDLVELLDSRPVDHA |
| Cb07 | CBG07924 | MSCPSESASAFRLRPVGSLFFLNRPHEKRAFDSLAGSGFGAFNKRAFDTMAGSGFSGFDKRAFDSLAGSGFGAFNKRAFDSLAGSGFSGFDKRAFDSLAGQGFTGFEKRAFDTVSTSGFDDFKL |
| Ce04 | CC4.2 | MPSSSSSSSFFAAVLLVIVMMSTVESAAVRLRPVGSLFFLNRPHEKRAFDSLAGSGFDNGFNKRAFDSLAGSGFGAFNKRAFDSLAGSGFGAFNKRAFDSLAGSGFSGFDKRAFDSLAGQGFTGFEKRAFDTVSTSGFDDFKL |
| As05 | ASU_05655 | MVSHFSYSSTLFKLASLTLILQFISATSISRSKDAKKPSLDILEGAGFSPLKKRALDTLEGSGFGFSKRALDDLEGVGFGGMLRKRALDSLEGTDFGLKKRALDYLEGSGFGLMKRAFDAIEGADIGFHKRALDMLEGSGFGLKKRALDSLEGTGFGLKKRALDSLDGAGFGFDKRALDVLEGTDFGFNKRALDDLEGAGFGLKKRALDAMEGAGFGFDKRALDSMEGTGFGFHKRALDGLEGDGFGFTKKALDSLEGTDFDIVTHVVDTTDGTSVMKI |
| Bx06 | BXY_0652000.1 | MTVHSRICLSAALFVMFCSAFVSSAVVVRLPVAPTRSVFFTPALFTKRALDALEGSDFGLKRKRALDSLEGSDFGLRKRALDSLEGSDFGLRKRALDSLEGADFGLKKRALDSLEGADFGLKKRALDSLEGADFGLRKRALDSLEGADFGLRKRALDSLEGSDFGLKKRALDALEGTGFGFDKRALDMLEGSDFGLKKRALDSLEGADFGLKKRALDSLEGSDFGLRKRALDSLEGSDFGLKKRSPIARFYATGDDIRKLNDLKTQLEVELRRRLEKEAEA |
| Sr00 | SRAE_2000443500 | MMTKSYCISKNFLRLFLTFLICSSVISAYYPEYGLRRQMRALDSIEGSDFGFKKRALDSLEGEGFGLKKRALDSIEGSDFGFKKRALDSLEGEGFGLKKRALDSIEGSDFGFKKRALDSLEGEGFGLKKRNSKFGNRYLAFLRYH |
| Bm29 | Bm2988 | MLSLILLVLALGEFNDAKSKFDEIKKMRDTIKGSRFRAKQSLNSIDGSEFDGLGGIRIRIEKRSLDALQGEGFGMKKRALDALEGEGFGMKKRALDALEGEGFGMKKRALDALEGEGFGMKKRALDALEGEGFGMKKRALDALEGEGFGMKKRALDALEGEGFGMKKRALDALEGEGFGMKKRALDALEGEGFGMKKRALDALEGEGFGMKKRALDALEGEGFGMKKRALDALEGEGFGMKKRALDALEGEGFGMKKRALDALEGEGFGIDKRVLDALEGAGLRMNRPLKSSDSRTKGFH  MNRFSTNGRHSHPHLTKF |
| Sc20 | L596_g20527.t1 | MLQSLSCCCVFSICVLVASALTQSATHGPSAIDELRKSVQLMKSRHAIDSLEGTGFDSLRKRALDSFEGDGFGMKKRALDILDGNDFGMKKRALDYLEGGDFGMKKRALDILDGNDFGMKKRALDYLEGGDFGMKKKRALDYLEGGDFGMKKRALDYLEGGDFGMKKRALDILDGNDFGMKKRALDYLEGGDFGMKKKRALDILDGNDFGMKKRALDYLEGGDFGMKKRALDILDGNDFGMKKRALDYLEGGDFGMKKRSSQQLLGRLRGKRDRTLAALNRNQLRAYNEEIRRKMRNA |
| Sf89 | L889_g18568.t1 | MTNFLLHNVLASREGAFPPVTPPHSVEEITRATTMTQRRFAYSSFVSISVLLAVTAAATVTAGPQEIDELRKSVALLKSRHALDALEGNGFTSLRKRALDSFEGDGFGMRKRALDSLEGMEFGMKKRALDSLEGMEFGMKKRALDYLEGGMEFGMKKRALDSLEGMEFGMKKRALDYLEGGDFGMKKRALDYLEGGDFGMKKRALDYLEGGDFGMKKRALDSLEGMEFGMKKRALDSLEGMEFGMKKRALDYLEGGDFGMKKRALDYLEGGDFGMKKRALDSLEGMEFGMKKRTYRLLGRLRGKRAFSSLARNQIRAFNEQMRRKMATMQQA |
| Cb00 | CBG00246 | MLHPIVLLLVASSAVYAARPRRALDGLDGSGFGFDKRALNSLDGAGFGFEKRALNSLDGQGFGFEKRALDGLDGMGFGFDKRALNSLDGAGFGFEKRALDGLDGAGFGFDKRALNSLDGQGFGFEKRALNSLDGAGFGFEKRALDGLDGAGFGFDKRALNSLDGAGFGFEKRALDGLDGAGFGFDKRALNSLDGQGFGFDKRSSFKHQSSKLRSVFRNLRGFNKQH |
| Ce1a | D1009.4a | MLHLIVLLVALSSAVTAGRPRRALDGLDGSGFGFDKRALNSLDGAGFGFEKRALNSLDGQGFGFEKRALDGLDGAGFGFDKRALNSLDGAGFGFEKRALDGLDGSGFGFDKRALNSLDGAGFGFEKRALNSLDGAGFGFEKRALDGLDGAGFGFDKRALNSLDGAGFGFEKRALDGLDGAGFGFDKRALNSLDGNGFGFDKRTFKHSSNKLRSVFRNLKGFKQH |
| Ce1b | D1009.4b | MLHLIVLLVALSSAVTAGRPRRALDGLDGSGFGFDKRALNSLDGAGFGFEKRALNSLDGQGFGFEKRALDGLDGAGFGFDKRALNSLDGAGFGFEKRALDGLDGAGFGFDKRALNSLDGAGFGFEKRALDGLDGAGFGFDKRALNSLDGNGFGFDKRTFKHSSNKLRSVFRNLKGFKQH |
| Pp00 | PPA00238 | MIAKHTLAVLLVAATVSSIEALRAKRALDGLDGAGFGFQKRALNSLDGDGFGFNKRALDGLDGAGFGFQKRALDGLDGEGFGFQKRALNSLDGDGFGFNKKKRALDGLDGEGFGFQKRALNSLDGDGFGFNKRALDGLDGEGFGFQKRAANRQQRIRMLRQFLF |
| Hc00 | HCOI00090300.t1 | MLFFLTRCVLVVGLSQLVCSEQDATHPPPALDDLEGNGFGGMKKFSSRRKRALDSLEGDGFGGLFKRSLDSLEGDGFGFDKRALNALDGTGFGFDKRALNSLEGTGFGFDKRSLNSIEGTGFGFDKRSLNSIEGTGFGFDKRSLNSIEGTGFGFDRRRRSLDSTEGTGFGYRRGKRTLPQITGTHPYLRLYKGKQSRGYKGDRSNPNGSNTYLHNN |
| Gp38 | GPLIN_000384700 | MAEAKNICRTKTPFSDFKLMVLLGIVLCSLFTPSTSIKESSRSSSLGRTGAIKVLALRPRRALDILESDDFGGFRKRALDVMDGDGFGSFEKRALDTLEGDDFMGLKRKRLNELEGDGFMGLDKRALDILDGDDFTGFSKRSEVNGGLRRELALLGKVQRQRQMRARRALDALEGNSFGFRR |
| Mh24 | MhA1_Contig2.frz3.gene24 | MISKILILFALCCICRPVNSSNNKNINSNSAQHNFRTHIIVGTEKELTNLFNSFHPKSSTTIIKRALDMLEGDDFIGMQKKRKRALDLMDGDGFSGFDKRALDMMEGDDFIGLKKKRRSSRFNKRALDILEGDDFVGMQKR |
| Mi06 | Minc06775 | MTYLTENFGIFRKFSKIMILFALCCICRLVNCSNNNNINSAQHNNLRITPILVGTEKELSTFFNSFHQHPKNTLIKRALDMLEGDDFIGMQKRKRALDLMEGDGFGGGFDRKRRALDMMEGDDFIGLKKRSNMLRKRALDILEGDDFVGMQKRALLM |

**Supplemental Table S4.** **The enriched GO terms of DEGs involved in plant defense responses(FDR<0.01).**

| Gene ID | Gene annotation | | | Symbol | FPKM | | logFC | FDR |
| --- | --- | --- | --- | --- | --- | --- | --- | --- |
|  |  |  |  |  | WT | MiISE6 |  |  |
| Jasmonic acid mediated signaling pathway (GO:0009867) | | | | | | | | |
| AT2G34600 | Jasmonate-zim-domain protein 7 | | | JAZ7 | 1.833895 | 53.73022 | 4.83581 | 3.43701E-39 |
| AT1G17380 | Jasmonate-zim-domain protein 5 | | | JAZ5 | 2.76473 | 36.34035 | 3.668618 | 8.60072E-20 |
| AT1G30135 | Jasmonate-zim-domain protein 8 | | | JAZ8 | 1.187913 | 17.52833 | 3.852163 | 7.33319E-31 |
| AT1G19180 | Jasmonate-zim-domain protein 1 | | | JAZ1 | 47.80905 | 255.6724 | 2.390854 | 2.25023E-13 |
| AT5G13220 | Jasmonate-zim-domain protein 10 | | | JAZ10 | 5.935934 | 24.60796 | 2.041292 | 3.99608E-20 |
| AT3G50060 | Myb domain protein 77 | | | MYB77 | 6.127976 | 37.21285 | 2.613987 | 8.36001E-22 |
| AT5G67300 | Myb domain protein r1 | | | MYBR1 | 33.84092 | 117.1346 | 1.784778 | 7.83948E-13 |
| AT1G72450 | Jasmonate-zim-domain protein 6 | | | JAZ6 | 31.34895 | 79.15571 | 1.31643 | 3.98518E-07 |
| AT1G74950 | Jasmonate-zim-domain protein 2 | | | JAZ2 | 27.9711 | 71.12022 | 1.32044 | 3.87359E-08 |
| AT1G18570 | Myb domain protein 51 | | | MYB51 | 26.66989 | 65.12215 | 1.292883 | 0.006868244 |
| AT5G44420 | Plant defensin 1.2 | | | PDF1.2 | 12.63338 | 3.460612 | -1.86814 | 0.00145846 |
| AT3G04720 | Pathogenesis-related 4 | | | PR4 | 1.589795 | 0.284558 | -2.48204 | 0.000189518 |
| Genes involved in defense response regulation(GO:0031347) | | | |  |  |  |  |  |
| AT1G80840 | WRKY DNA-binding protein 40 | | | WRKY40 | 16.05074 | 169.0437 | 3.380604 | 2.68841E-30 |
| AT4G11070 | WRKY DNA-binding protein 41 | | | WRKY41 | 0.215475 | 0.832402 | 1.949066 | 0.003861361 |
| AT5G49520 | WRKY DNA-binding protein 48 | | | WRKY48 | 3.102297 | 21.44206 | 2.765911 | 2.57093E-31 |
| AT4G31800 | WRKY DNA-binding protein 18 | | | WRKY18 | 21.97554 | 102.5326 | 2.209323 | 7.91435E-16 |
| AT4G12720 | MutT/nudix family protein | | | NUDT7 | 15.02772 | 55.27315 | 1.873469 | 1.32415E-17 |
| AT3G15210 | Ethylene responsive element binding factor 4 | | | ERF4 | 63.89478 | 177.5442 | 1.474038 | 5.53181E-08 |
| AT4G39030 | MATE efflux family protein | | | EDS5 | 4.947456 | 11.70742 | 1.236 | 0.000365638 |
| Response to salicylic acid stimulus(GO:0009751) | | | | | | | | |
| AT1G28480 | Thioredoxin superfamily protein | | | GRX480 | 6.536158 | 55.7258 | 3.048667 | 4.00836E-08 |
| AT1G80840 | WRKY DNA-binding protein 40 | | | WRKY40 | 16.05074 | 169.0437 | 3.380604 | 2.68841E-30 |
| AT3G61190 | BON association protein 1 | | | - | 4.05877 | 44.41178 | 3.446685 | 8.85731E-26 |
| AT4G31800 | WRKY DNA-binding protein 18 | | | WRKY18 | 21.97554 | 102.5326 | 2.209323 | 7.91435E-16 |
| AT3G52400 | syntaxin of plants 122 | | | SYP122 | 42.99888 | 203.2872 | 2.238808 | 6.78266E-14 |
| AT3G50060 | myb domain protein 77 | | | MYB77 | 6.127976 | 37.21285 | 2.613987 | 8.36001E-22 |
| AT5G62470 | myb domain protein 96 | | | MYB96 | 6.100616 | 25.73509 | 2.071354 | 1.80012E-28 |
| AT5G22570 | WRKY DNA-binding protein 38 | | | WRKY38 | 2.280752 | 9.12665 | 2.005442 | 3.37778E-06 |
| AT5G67300 | myb domain protein r1 | | | MYBR1 | 33.84092 | 117.1346 | 1.784778 | 7.83948E-13 |
| AT3G11820 | syntaxin of plants 121 | | | SYP121 | 29.42899 | 82.81214 | 1.488248 | 2.19912E-09 |
| AT2G40000 | ortholog of sugar beet HS1 PRO-1 2 | | | - | 97.40864 | 456.0174 | 2.245498 | 3.07682E-15 |
| AT1G18570 | myb domain protein 51 | | | MYB51 | 26.66989 | 65.12215 | 1.292883 | 0.006868244 |
| AT4G37260 | myb domain protein 73 | | | MYB73 | 67.3669 | 180.8132 | 1.427697 | 3.8193E-09 |
| AT5G37260 | Homeodomain-like superfamily protein | | | - | 6.735943 | 3.287833 | -1.01443 | 5.26E-05 |
| Response to auxin stimulus(GO:0009733) | | | | | | | | |
| AT5G37770 | EF hand calcium-binding protein family | | | TCH2 | 10.91358 | 89.97975 | 3.039514 | 6.03144E-44 |
| AT4G11280 | 1-aminocyclopropane-1-carboxylic acid (acc) synthase 6 | | | ACS6 | 15.01691 | 91.88069 | 2.598273 | 1.64149E-13 |
| AT5G62470 | myb domain protein 96 | | | MYB96 | 6.100616 | 25.73509 | 2.071354 | 1.80012E-28 |
| AT5G54490 | pinoid-binding protein 1 | | | PBP1 | 6.94835 | 27.84776 | 2.021774 | 4.66089E-05 |
| AT3G12830 | SAUR-like auxin-responsive protein family | | | - | 3.698508 | 11.36769 | 1.645084 | 5.65914E-07 |
| AT4G37260 | myb domain protein 73 | | | MYB73 | 67.3669 | 180.8132 | 1.427697 | 3.8193E-09 |
| AT5G59780 | myb domain protein 59 | | | MYB59 | 14.6664 | 6.304026 | -1.22501 | 1.78965E-08 |
| AT5G37260 | Homeodomain-like superfamily protein | | | - | 6.735943 | 3.287833 | -1.01443 | 0.004361778 |
| AT4G38860 | SAUR-like auxin-responsive protein family | | | - | 5.832595 | 2.090564 | -1.47983 | 7.528E-05 |
| AT3G59900 | auxin-regulated gene involved in organ size | | | - | 17.21423 | 6.626947 | -1.33672 | 0.003641987 |
| AT4G34790 | SAUR-like auxin-responsive protein family | | | - | 3.719499 | 1.090201 | -1.7655 | 7.68352E-05 |
| AT1G29430 | SAUR-like auxin-responsive protein family | | | - | 4.518414 | 1.045475 | -2.10663 | 6.8507E-08 |
| Genes involved in calcium signaling pathway(GO:0005516; GO:0004683) | | | | | | | | |
| AT1G76640 | Calcium-binding protein CML39 | | | CML39 | 0.666234 | 4.885679 | 2.827158 | 1.6829E-06 |
| AT5G42380 | Calcium-binding protein CML37 | | | CML37 | 2.303882 | 52.44411 | 4.508121 | 3.31181E-44 |
| AT3G01830 | Probable calcium-binding protein CML40 | | | CML40 | 0.14458 | 2.665425 | 4.235524 | 3.04382E-16 |
| AT1G76650 | Calcium-binding protein CML38 | | | CML38 | 8.095486 | 94.51974 | 3.511682 | 5.12322E-17 |
| AT5G37770 | Calcium-binding protein CML24 | | | CML24 | 10.91358 | 89.97975 | 3.039514 | 6.03144E-44 |
| AT3G57530 | Calcium-dependent protein kinase 32 | | | CPK32 | 14.51999 | 74.56302 | 2.35089 | 6.17186E-26 |
| AT5G66210 | Calcium-dependent protein kinase 28 | | | CPK28 | 14.00694 | 70.52446 | 2.314423 | 8.56353E-20 |
| AT2G43290 | Calcium-binding EF-hand family protein | | | CML5 | 18.1328 | 88.59631 | 2.269875 | 3.7429E-26 |
| AT5G47910 | Respiratory burst oxidase homolog protein D | | | RBOHD | 29.17498 | 102.2822 | 1.790055 | 5.98502E-06 |
| AT1G66400 | Probable calcium-binding protein CML23 | | | CML23 | 3.385005 | 14.21229 | 2.079782 | 1.07169E-13 |
| AT3G17690 | Putative cyclic nucleotide-gated ion channel 19 | | | CNGC19 | 0.560903 | 2.345451 | 2.059324 | 1.39869E-11 |
| AT1G76040 | Calcium-dependent protein kinase 29 | | | CPK29 | 4.854515 | 14.3083 | 1.545489 | 5.9025E-12 |
| AT3G25600 | Probable calcium-binding protein CML16 | | | CML16 | 11.96999 | 38.22962 | 1.682952 | 1.24855E-11 |
| AT2G41100 | Calmodulin-like protein 12 | | | CML12 | 43.73411 | 91.46805 | 1.074866 | 0.00129945 |
| Genes involved in ubiquitination proteasome pathway(GO:0016567) | | | | | | | | |
| AT1G60190 | ARM repeat superfamily protein | | PUB19 | | 0.179564 | 4.788732 | 4.693922 | 2.47092E-14 |
| AT2G35930 | Plant U-box 23 | | PUB23 | | 4.214722 | 38.46382 | 3.183825 | 4.88172E-27 |
| AT2G44578 | RING-U-box superfamily protein | | - | | 2.588936 | 13.6704 | 2.415538 | 4.40193E-05 |
| AT1G20823 | RING-U-box superfamily protein | | - | | 4.174495 | 31.95405 | 2.935395 | 3.97507E-34 |
| AT3G52450 | Plant U-box 22 | | PUB22 | | 6.149499 | 27.54045 | 2.153482 | 1.09441E-10 |
| AT3G10815 | RING-U-box superfamily protein | | - | | 1.889595 | 7.092052 | 1.908931 | 1.10693E-08 |
| AT4G15975 | RING-U-box superfamily protein | | - | | 2.240686 | 8.065202 | 1.859098 | 1.37327E-07 |
| AT1G10560 | Plant U-box 18 | | PUB18 | | 0.637531 | 2.024188 | 1.638347 | 1.69254E-05 |
| AT2G21500 | RING-U-box superfamily protein | | - | | 7.160464 | 18.47446 | 1.342776 | 1.09952E-08 |
| AT4G36550 | ARM repeat superfamily protein | | - | | 3.572082 | 9.95477 | 1.462839 | 7.46309E-11 |
| AT5G67340 | ARM repeat superfamily protein | | - | | 5.649795 | 9.817612 | 1.178144 | 0.002296609 |
| AT3G11840 | Plant U-box 24 | | PUB24 | | 3.083249 | 6.491224 | 1.062841 | 0.001092054 |
| AT1G26800 | RING-U-box superfamily protein | | - | | 10.36374 | 4.927846 | -1.04164 | 0.007513332 |
| AT1G14200 | RING-U-box superfamily protein | | - | | 14.71125 | 6.185762 | -1.21425 | 0.000710067 |
| AT1G49210 | RING-U-box superfamily protein | | - | | 3.327278 | 0.595215 | -2.47757 | 1.33365E-06 |
| AT4G28270 | RING membrane-anchor 2 | RMA2 | | | 6.815862 | 3.239509 | -1.07927 | 0.002658569 |
| AT4G03510 | RING membrane-anchor 1 | | RMA1 | | 10.28842 | 4.802533 | -1.09915 | 0.00571955 |
| Genes related to cell wall modification(GO:0042545) | | | | | | | | |
| AT3G45960 | expansin-like A3 | | | EXLA3 | 0.733102 | 18.00623 | 4.629249 | 4.02737E-45 |
| AT3G45970 | expansin-like A1 | | | EXLA1 | 9.646493 | 81.30135 | 3.057966 | 1.92313E-21 |
| AT4G38400 | expansin-like A2 | | | EXLA2 | 4.203884 | 18.44121 | 2.116415 | 1.20682E-09 |
| AT1G69530 | expansin A1 | | | EXLA1 | 71.68371 | 35.59864 | -1.01226 | 0.000168943 |
| AT4G30280 | xyloglucan endotransglucosylase-hydrolase 18 | | | XTH18 | 17.99377 | 140.0172 | 2.952691 | 1.04971E-21 |
| AT5G57560 | Xyloglucan endotransglucosylase-hydrolase family protein | | | TCH4 | 52.39179 | 397.8505 | 2.932393 | 6.04705E-23 |
| AT4G30290 | xyloglucan endotransglucosylase-hydrolase 19 | | | XTH19 | 9.793053 | 40.54704 | 2.056187 | 1.03781E-18 |

|  |  |  |  |  |  |  |
| --- | --- | --- | --- | --- | --- | --- |

Log10

-1 0 1 2

**Supplemental Table S5.** **The enriched KEGG terms of DEGs (P<0.05).**

| Items_Details | ID | Number of genes | P-value | Genes |
| --- | --- | --- | --- | --- |
| Plant-pathogen interaction | ath04626 | 18 | 3.71E-20 | CPK29,CML39,AT3G59350,CPK32,KCS1,CML5,CML38,CNGC13,CNGC19,CML23,CML12,CML16,CPK28,CML40,RBOHD,AT3G10300 ,CML24,CML37 |
| alpha-Linolenic acid metabolism | ath00592 | 6 | 0.000109 | AOC3,4CLL5,OPR3,CYP74A,LOX4,LOX3 |
| Plant hormone signal transduction | ath04075 | 13 | 0.000307 | TIFY10B(JAZ2),SAUR72,TIFY11A(JAZ5),NPR3,AT3G09870,TIFY10A(JAZ1),JAZ10,XTH22,IAA4,TIFY11B(JAZ6),GH3.1,AT4G34770,AT4G38860 |
| Carotenoid biosynthesis | ath00906 | 4 | 0.001697 | CYP707A3,CCD4,NCED3,CYP707A1 |
| Starch and sucrose metabolism | ath00500 | 10 | 0.002147 | PME41,BAM3,PME20,GAE1,TPPD,PME7,PME25,BGLU46,BAM1,TPPG |
| Glycerolipid metabolism | ath00561 | 5 | 0.015362 | ATGPAT3, AT1G73480,DGK2,DGK1,GPAT2 |
| Diterpenoid biosynthesis | ath00904 | 3 | 0.0341132 | CYP82G1,GA2OX2,GA2OX6 |
| Linoleic acid metabolism | ath00591 | 2 | 0.036101 | LOX4,LOX3 |

**Supplemental Figure S1. Venn diagram of the transcriptome in Avir-1, Avir-2 and Avir-3 of *M. incognita*. The number (13,398) in the overlapping area represents the common genes among Avir-1, Avir-2 and Avir-3.**


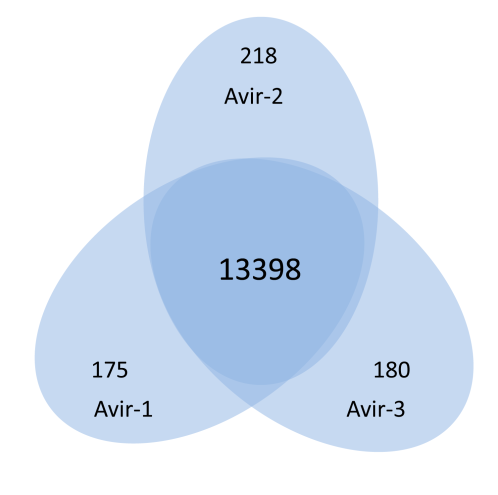


**Supplemental Figure S2. The structure of domains of MiISE6.**

**
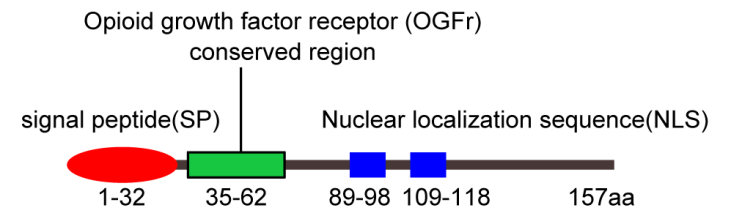
**

**Supplemental Figure S3.** **Original picture of RT-PCR assays used for checking the expression of MiISE6 and Actin2 in three independent overexpressing lines.**

**
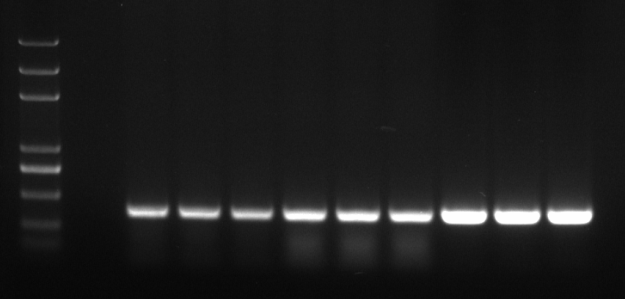
**

250bp

MiISE6

**
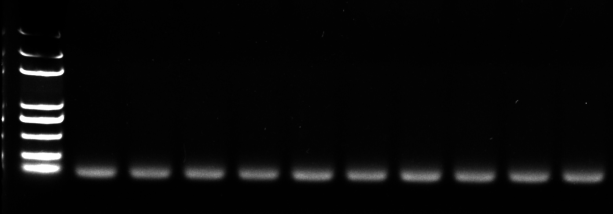
**

100bp

Actin2

**Supplemental Figure S4.** Root galls development of control(WT) and MiISE6 transgenic Line (T_1,T_2 and T_8).


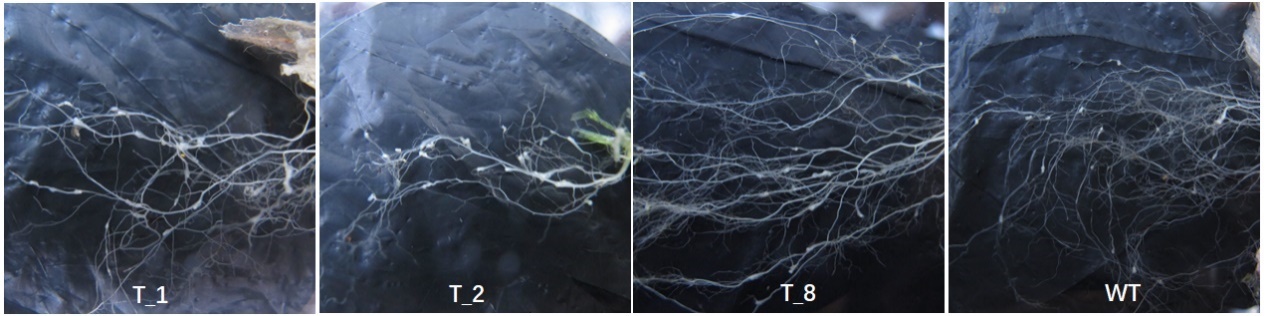


**Supplemental Figure S5.** Statistically significant differences between treatments (WT-T_1, WT-T_2 and WT-T_8) were determined by independent samples t-test (P < 0.05) with SPSS. Following figures showed results of t-tests (WT with T_1, T_2 and T_8, respectively). The results showed that there was highly significant difference in the number of females between WT plants and the transgenic plants (P<0.01).

WT-T_1


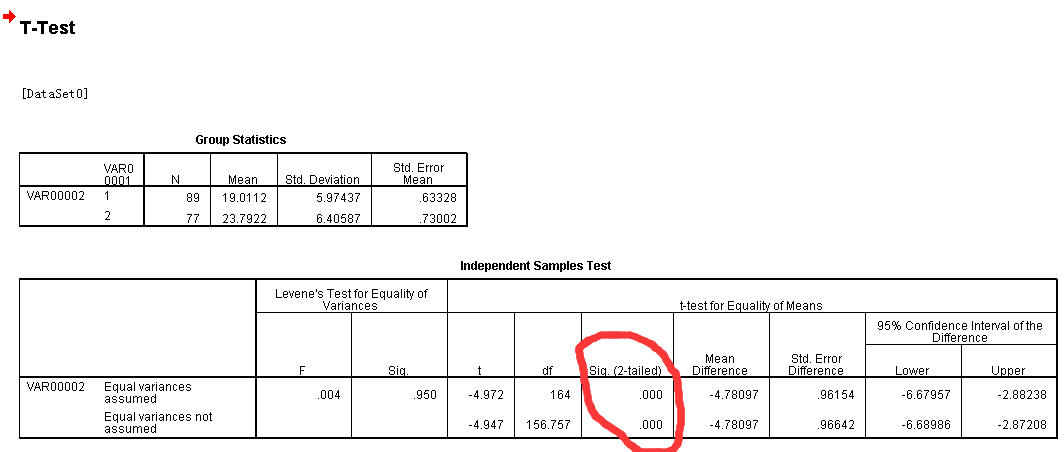


WT-T_2


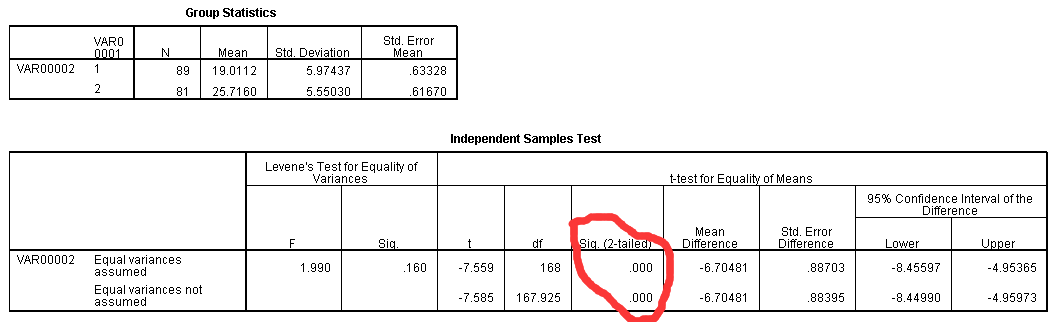


WT-T_8


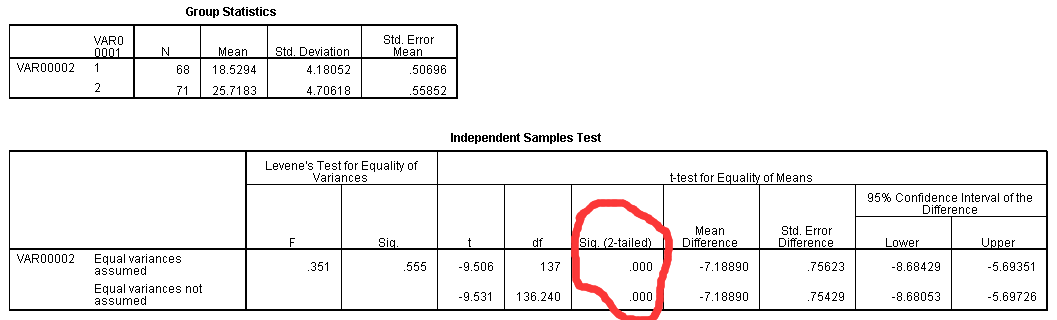


**Supplemental Figure S6. RT-PCR of the single-stranded CHAS intron of MiISE6 dsRNA hairpins conformed the expression of RNAi1, RNAi5 and RNAi11 constructs in independent transgenic Arabidopsis lines.**

RNAi1

RNAi5

RNAi11

WT


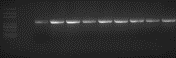


1000bp

**Supplemental Figure S7.** Root galls development of control (WT) and three host-derived RNAi lines of MiISE6 (RNAi1, RNAi5 and RNAi11).


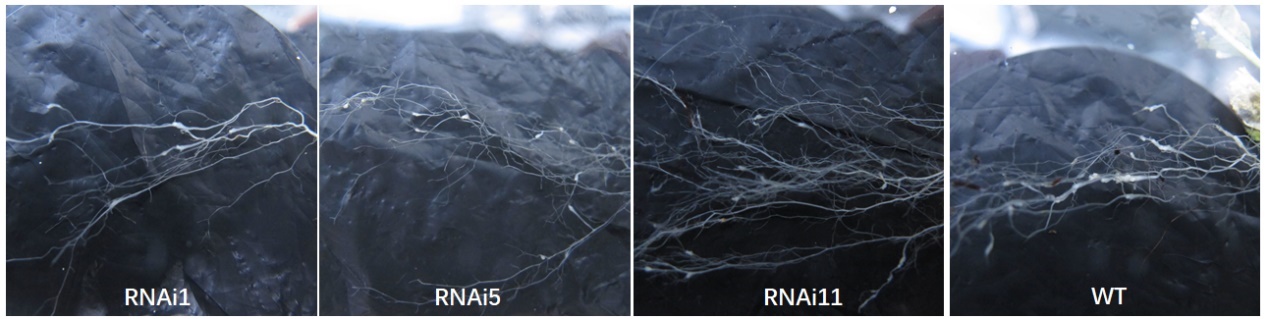


**Supplemental Figure S8.** Statistically significant differences between treatments (WT-RNAi1, WT-RNAi5 and WT-RNAi11) were determined by independent samples t-test (P < 0.05) with SPSS. Three RNAi lines (RNAi1, RNAi5, RNAi11), with at least 15 plants for each treatment, and three independent experiments. The results showed that the mean values of RNAi1 were significantly different from the control (P<0.01).

WT-RNAi1


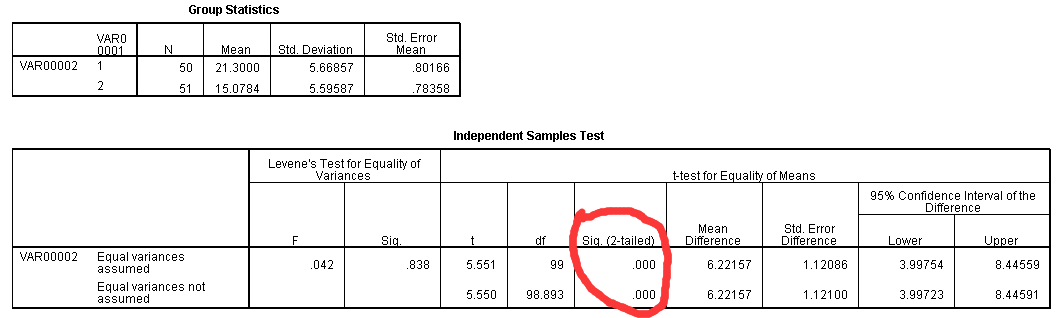


WT-RNAi5


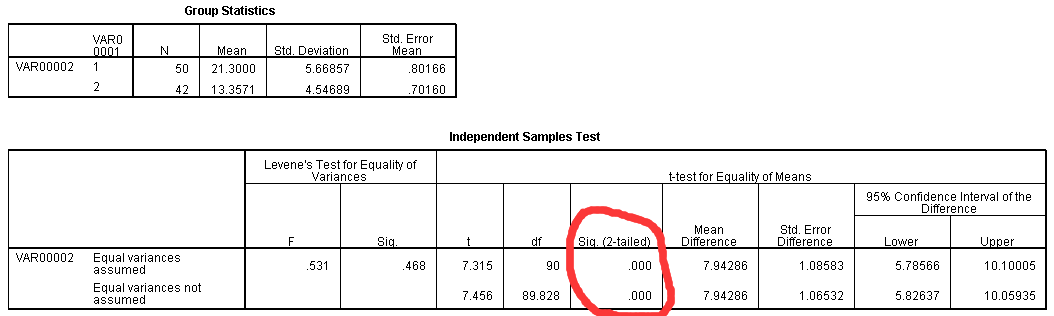


WT-RNAi11


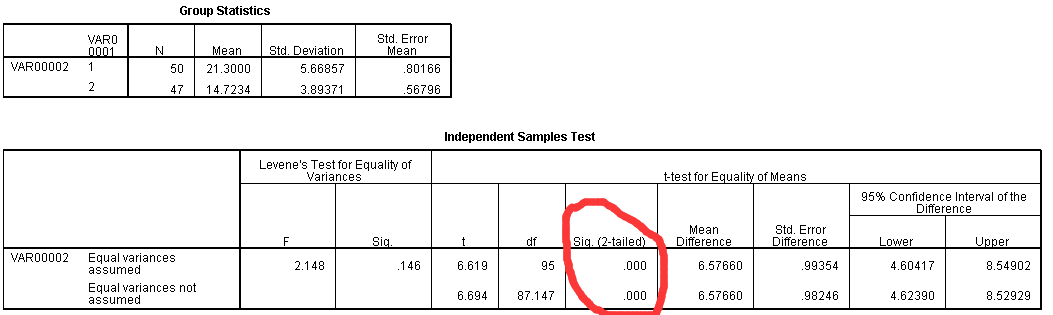

Supplement: Supplementary file 5 [file Data_Sheet_5.doc]
